# Italian guidelines for the treatment of type 2 diabetes

**DOI:** 10.1007/s00592-022-01857-4

**Published:** 2022-03-15

**Authors:** Edoardo Mannucci, Riccardo Candido, Lina delle Monache, Marco Gallo, Andrea Giaccari, Maria Luisa Masini, Angela Mazzone, Gerardo Medea, Basilio Pintaudi, Giovanni Targher, Marina Trento, Giuseppe Turchetti, Valentina Lorenzoni, Matteo Monami, for Società Italiana di Diabetologia (SID) and Associazione Medici Diabetologi (AMD)

**Affiliations:** 1 Diabetology, Azienda Ospedaliero-Universitaria Careggi, Careggi Hospital, University of Florence, Largo Brambilla, 50134 Florence, Italy; 2 ASUI, Trieste, Italy; 3 FAND, Milan, and FederDiabete Lazio, Rome, Italy; 4 Endocrinology and Metabolic Diseases, Hospital of Alessandria, Alessandria, Italy; 5 Endocrinology and Metabolic Diseases, Gemelli Hospital, Catholic University of Rome, Rome, Italy; 6 University of Florence, Firenze, Italy; 7 Retired, Formerly Diabetology, San Martino Hospital, Genova, Italy; 8 Società Italiana Di Medicina Generale (SIMG), Firenze, Italy; 9 Niguarda Ca’ Granda Hospital, Milan, Italy; 10 Endocrinology, Diabetology and Metabolic Diseases, University of Verona, Verona, Italy; 11 Laboratory of Clinical Pedagogy, University of Turin, Torino, Italy; 12 Scuola Superiore S. Anna, Pisa, Italy

## Abstract

**Aim:** This guideline is aimed at providing a reference for the pharmacological and non-pharmacological treatment of type 2 diabetes in adults.

**Methods**: These recommendations apply to outpatients, either in primary care or at specialist referral. Prior cardiovascular events, heart failure, renal disease, hypoglycemic risk and other conditions affecting life expectancy have been considered as factors capable of modifying treatment strategies. The following areas have been assessed: therapeutic goals, nutritional therapy, physical exercise, educational programs, pharmacological treatment, glucose monitoring. This guideline has been developed following the methods described in the Manual of the National Guideline System (http://www.snlg-iss.it). For each question, the panel nominated by the Società Italiana di Diabetologia (SID) and Associazione Medici Diabetologi (AMD) identified potentially relevant outcomes, which were then rated for their impact on therapeutic choices. Only outcomes classified as “critical” were considered in the systematic review of evidence and in the formulation of recommendations.

**Results:** The present guideline contains recommendations on the following clinical aspects of type 2 diabetes: 1) treatment targets; 2) nutritional therapy; 3) physical exercise; 4) educational therapy; 5) pharmacological treatment (for patients with and without previous cardiovascular disease); and 6) glycemic monitoring.

**Conclusions:** The present guideline is directed to physicians, nurses, dietitians and educators working in Diabetes specialist clinics; general practitioners; nurses and dietitian working in territorial services or private offices; and patients with diabetes.

**LISTS OF ABBREVIATIONS AND ACRONYMS**

LG: Linea Guida

AMD: Associazione Medici Ospedalieri

SID: Società Italiana di Diabetologia

PICOS: Population, Intervention, Comparison, Outcome, Study type

MNT: Medical Nutrition Therapy

NPH: Neutral Protamine Hagedorn

AMSTAR

MH-OR: Mantel–Haenszel Odds Ratio

WMD: Weighted mean difference

GRADE: Grades of Recommendation, Assessment, Development, and Evaluation

EtD: Evidence to Decision

**GUIDELINE DEVELOPMENT TEAM**

***Coordinator*:** Edoardo Mannucci, diabetologist.

***Panel members*:** Riccardo Candido, diabetologist; Lina delle Monache, diabetic patient; Marco Gallo^4^, diabetologist; Andrea Giaccari, diabetologist; Maria Luisa Masini, dietitian; Angela Mazzone, nurse; Gerardo Medea, general practitioner; Basilio Pintaudi, diabetologist Giovanni Targher, diabetologist; Marina Trento, pedagogist; Giuseppe Turchetti, economist.

***Evidence Review Team*:** Matteo Monami, Valentina Lorenzoni

***External reviewers*:** Giampaolo Fadini^1^, Antonio Nicolucci^2^, Gianluca Perseghin^3^

^1^Department of Medicine, University of Padova; ^2^Coresearch, Pescara; ^3^Metabolic Medicine, Policilinico di Monza, Bicocca University of Milan

**CONFLICTS OF INTEREST**

The assessment of interests of members of the Guideline development team is aimed at determining conflicts of interest for each question and the actions needed for their management in the process of elaboration of the Guideline. The assessment is based on the policy of the Istituto Superiore di Sanità for the management of conflicts of interest in the development of Guideline^1^. Each interest is assessed for its nature, type, relevance for the content of the Guideline, economic value, timing and duration. The assessment includes the following information which can be of help in determining the extent to which the competing interest could reasonably affect the expert’s position: type of interest; relevance for the content of the guideline; timing and duration; and position of the expert in the organization (in case of institutional interests).

With respect to type of potentially competing interests, these include:

With respect to type of potentially competing interests, these include:

1. Economic interests, i.e., financial relationships with organizations directly producing goods or services relevant for the guideline topic. Economic interests include any monetary transaction or value related to payments for services, property shares, stock options, patents and royalties. Relevant interest can be personal, related to family members or institutional (i.e., related to the organization in which the expert works).
2. Indirect interests, such as career advancement, social position and personal beliefs.

Interests considered can be:

1. Economic interests, i.e., financial relationships with organizations involved in products or services relevant for the subject of the guideline, including any direct payment for services, property shares, stock options, and patents or copyright royalties).

Economic interests can be either:

- personal economic interest, i.e., related to a personal financial benefit;
- familial economic interest, i.e., related to the income of family members;
- institutional economic interests, i.e., related to benefits for the institution in which the subject works.

2. Intellectual interests, i.e., benefits for career advancement and social status.

Both economic and intellectual interests can be specific (i.e., directly related to the subject of the guideline) or aspecific (when they are not related to the content of the guideline).

Any reported potentially conflicting interest is classified as:

- Level 1 (minimal or not relevant): no action needed
- Level 2 (potentially relevant): this can be managed either with
  - full participation to the development of the guideline with public disclosure of the conflict of interest at the end of the recommendation related to the interest;
  - exclusion of the subject with the competing interest form the discussion of those recommendations possibly influenced by the competing interest.
- Level 3 (relevant): this can be managed with the exclusion of the subject with the competing interest from the discussion of possibly affected recommendation, or with the total exclusion of the subject with competing interest from the elaboration of the guideline.

**DECLARATION OF POTENTIAL CONFLICTS OF INTEREST**

All members of the panel and of the evidence review team compiled annually a declaration of potential conflicts of interest, which were collectively discussed to determine their relevance. In all cases, the reported conflicts were considered minimal or irrelevant (Level 1); therefore, all components of the panel and of the evidence review team participated to the elaboration of all recommendations.

***Panel members:*** Edoardo Mannucci received fees for training activities from Mundipharma and speaking fees from Abbott, Eli Lilly e Novo Nordisk; Riccardo Candido received consulting fees from Boehringer Ingelheim, Eli Lilly, Merck, Menarini and Roche, and speaking fees from Abbott, Eli Lilly, Mundipharma, Novo Nordisk and Sanofi; Andrea Giaccari received consulting fees from Abbott, AstraZeneca, Boehringer Ingelheim, Eli Lilly, Merck, Mundipharma, Novo Nordisk e Sanofi, and his Institution received research grants from Amgen and AstraZeneca; Gerardo Medea received consulting fees from AstraZeneca and Grunenthal; Basilio Pintaudi received consulting and/or speaking fees from Eli Lilly e Novo Nordisk; and Giovanni Targher received consulting fees from Novartis; Giuseppe Turchetti received speaking fees from Eli Lilly, and his Institution received research grants from Merck. Lina Delle Monache, Marco Gallo, Maria Luisa Masini, Angela Mazzone and Marina Trento have no interest to declare.

***Evidence review team members:*** Matteo Monami receives speaking fees from Sanofi; Valentina Lorenzoni has no interest to declare.

***External reviewers:*** Gian Paolo Fadini received research grants from Mundipharma, consulting fees from Abbott, Boehringer, Novo Nordisk and Lilly, and speaking fees from Abbott, Novo Nordisk, Sanofi, Boehringer e AstraZeneca; Gianluca Perseghin received consulting fees from AstraZeneca, Boehringer Ingelheim, Eli Lilly, Merck, Novo Nordisk, PicDare; and Antonio Nicolucci received research grants from Sanofi and Novo Nordisk.

**FINANCIAL SUPPORT**

No external financial support was collected for the development of this guideline. Travel expenses for panel meeting were paid for by Società Italiana di Diabetologia. Members of panel and evidence review team did not receive any payment for their work in developing the guideline.

**AIMS OF THE GUIDELINE**

Type 2 diabetes is the most common form of diabetes; its prevalence is rapidly increasing, with a relevant impact on public health. People with type 2 diabetes (over 3 million in Italy) show increased risks of hospitalization, disability and mortality with a yearly cost exceeding 20 billion Euros3.

In Italy, the care of patients with type 2 diabetes is provided by a capillary network of specialist clinics and general practitioners, which warrants a good quality of healthcare. However, some areas still need to be improved: A fraction of patients does not reach therapeutic targets and the management of pharmacological therapy is widely heterogeneous. This heterogeneity is partly determined by the fast development of therapeutic options and clinical evidences; the timely synthesis of those evidences in the format of clinical recommendations and their dissemination among physicians is objectively difficult. The two main dialectological societies in Italy formulated joint guidelines on the management of diabetes in 20,184, without participation of other healthcare professionals involved in the care of diabetes. In addition, other guidelines 5–7 formulated in different organizational contexts are often used by Italian healthcare providers.

This guideline is aimed at providing a reference for pharmacological and non-pharmacological treatment of type 2 diabetes in adults (age of 18 years or more).

Recommendations are designed as indications for healthcare professionals in charge of diabetes treatment, primarily based on clinical needs of people with diabetes and considering the existing organization of healthcare. These recommendations apply to outpatients, either in primary care or at specialist referral. Prior cardiovascular events, heart failure, renal disease, hypoglycemic risk and other conditions affecting life expectancy will be considered as factors capable of modifying treatment strategies.

The following areas will be assessed: therapeutic goals, nutritional therapy, physical exercise, educational programs, pharmacological treatment, glucose monitoring. All the interventions considered are usually reimbursed, with some regional differences for glucose monitoring devices and nutritional therapy. Recommendations will be formulated on the basis of available evidence, independent of current reimbursement policies.

The guideline is directed to physicians, nurses, dietitians and educators working in Diabetes specialist clinics; general practioners; nurses and dietitian working in territorial services or private offices; patients with diabetes. During the development of the guideline, available resources will be considered, verifying the effects of each recommendation on the organization of care and collecting cost-efficacy and cost-utility data whenever possible.

The implementation of the guideline will be pursued through their dissemination, performed by:

1) Scientific societies, using their websites and official journals and organizing specific activities of continuous medical education; 2) regional healthcare systems.

**METHODS FOR GUIDELINE DEVELOPMENT**

The guideline was developed following the methods described in the Manual of the National Guideline System (http://www.snlg-iss.it).

***Clinical questions***

Each recommendation answers a clinical question, formulated by the panel using the PICOS framework.

**Selection of outcomes**

For each question, the panel identified potentially relevant outcomes, which were then rated for their impact on therapeutic choices using a 9-point scale:

*0–3 points*: outcomes of limited relevance

*4–6 points*: important, but not critical outcomes

*7–9 points:* critical outcomes.

Only outcomes classified as “critical” were considered in the systematic review of evidences and in the formulation of recommendations. A complete list of outcomes with their scores, for each recommendation, is reported in Appendix.

**Evidence review and assessment of quality of evidence**

A systematic review for critical outcomes for each question was performed on the following databases:

- Cochrane Database of Systematic Reviews (Wiley)
- Cochrane Central Register of Controlled Trials (Wiley)
- MEDLINE (OVID)
- Embase (OVID)
- Clinicaltrials.gov

For pharmacoeconomic evidence, only Medline was searched, retrieving only studies assessing the different interventions for glucose control.

Specific search strategies were used for each database, as specified in each chapter of Appendix. Searches for pharmacoeconomic studies were limited to the last 10 years, whereas no time limits were imposed for all the other searches. Only items in English were considered. References of retrieved items were searched for further studies meeting inclusion criteria.

The systematic review was performed through the following steps:

- 1. Selection of potentially eligible studies obtained with the initial search, on the basis of title and abstract, for retrieval as full text;
- 2. Identification among retrieved full-text items of relevant studies, on the basis of a priori inclusion and exclusion criteria;
- 3. Critical assessment of the risk of bias using validated instruments (i.e., AMSTAR 2^8^ for systematic reviews and the Cochrane collaboration tool^9^ for randomized trials).
- 4. Extraction of the main characteristics of selected studies (population enrolled, considered outcomes, results), summarized in tables.
- 5. Quantitative synthesis for each outcome, calculating MH-OR for categorical outcomes and WMD for continuous variables, both with 95% confidence intervals. The main analysis was always performed with random effects models, whereas fixed effects models, when used, were considered only for sensitivity analyses;
- 6. Assessment of heterogeneity (I^2^) and of publication bias (Funnel plot);
- 7. The overall quality and strength of available evidence for outcomes selected by the panel were rated using the GRADE^10^ criteria.
- 8. Synthesis of results, using the GRADEPro Guideline Development tool (https://gradepro.org), with the frameworks EtD^11^, which summarize results of systematic reviews for problem priority, desired and undesired effects of treatments, strength of available evidence, values and preferences of stakeholders, economic resources needed, equity, acceptability and feasibility of interventions.

Statistical analyses were performed with RevMan 5.0 (https://training.cochrane.org/online-learning/core-software-cochrane-reviews/revman/revman-5-download) and MetaXL (http://epigear.com/index_files/metaxl.html) for traditional and network meta-analysis.

For pharmacoeconomic studies, relevant records were selected on the basis of title and abstract for full text retrieval. Due to the geographical and methodological heterogeneity of retrieved studies, no formal meta-analysis was performed; methods and results were summarized in tables, including type of analysis, context, year(s) to which costs were referred, efficacy, cost-efficacy and cost-utility, main conclusions.

**Development of recommendations**

The guideline panel examined and discussed, for each clinical question, EtD frameworks, tables of evidence and summaries of results (forest plots of meta-analyses). Recommendations were formulated on the basis of results of available studies and quality of evidence. Disagreements were resolved through collective discussion.

**External review**

The panel identified three external reviewers, chosen among Italian healthcare professionals with a specific experience of clinical research in diabetes, with known methodological skills, who had published at least 150 peer-reviewed original articles on International medical journals and who had a h-index of at least 40. Members of the guideline panel and evidence review team, and current members of the Board of SID or AMD, were excluded.

External reviewers received a draft version of the guideline and provided their observations to the panel. The panel collectively discussed the points raised by the external reviewers, elaborating the amendments to the guideline and the response to reviewers.

**Guideline update**

Systematic reviews will be updated, using the same search strings, once every year, starting from the date of final approval of the guideline. The evidence review team and the guideline panel will verify whether new evidences will modify the risk/benefit ratio or the overall quality of evidences to the extent of modifying the formulation of a recommendation, of its strength or of the quality of evidence.

Once every year, the guideline panel will verify the need to modify, update, add or remove clinical questions, and the opportunity of modifying the outcomes of interest and their relative relevance. In case of changes in clinical questions and/or critical outcomes, the whole process of evidence review and development of recommendation will be performed anew.

**INTERPRETATION OF RECOMMENDATIONS**

**Quality of evidence**

- HIGH: Highly reliable results. It is very unlikely that further studies modify the confidence in estimated effects.
- MODERATE: Moderately reliable results. It is possible that further studies modify the confidence in estimated effects.
- LOW: Results are still uncertain. Further research is needed for a reliable assessment of positive and negative effects of the intervention.
- VERY LOW: Available data are not reliable, and estimates of effects should be considered with caution.

**Strength of recommendations**

*Strong recommendation*

- for clinicians: the majority of patients must receive the recommended intervention;
- for patients: almost all properly informed patients follow the recommendation and only a small fraction choses different options;
- for policy makers: the recommendation can be used for planning the use of available resources.

*Weak recommendation*

- for clinicians: the final choice should include a careful consideration of patients’ values and preferences;
- for patients: the majority of properly informed patients follow the recommendation, but a minority choses different options;
- for policy makers: a discussion involving stakeholder should be developed.

**SUMMARY OF RECOMMENDATIONS**

1. **Treatment targets**

*Strength of the recommendation: strong. Quality of evidence: low.*

**1.1 A target HbA1c between 49 mmol/mol (6.6%) and 58 mmol/mol (7.5%) is recommended for patients with type 2 diabetes treated with drugs capable of inducing hypoglycemia.**

*Strength of the recommendation: strong. Quality of evidence: low.*

**1.2.1. A target HbA1c below 53 mmol/mol (7%) is recommended for patients with type 2 diabetes treated with drugs which are not capable of inducing hypoglycemia.**

*Strength of the recommendation: weak. Quality of evidence: very low.*

**1.2.2. A target HbA1c of 48 mmol/mol (6.5%) or lower is suggested for patients with type 2 diabetes treated with drugs which are not capable of inducing hypoglycemia.**

2. **Nutritional therapy**

*Strength of the recommendation: weak. Quality of evidence: low.*

**2.1 Structured Medical Nutrition Therapy is suggested for the treatment of type 2 diabetes.**

*Strength of the recommendation: weak. Quality of evidence: low.*

**2.2. We suggest a balanced (Mediterranean) diet, rather than a low-carbohydrate diet, for the treatment of type 2 diabetes. **

3. **Physical exercise**

*Strength of the recommendation: weak. Quality of evidence: moderate.*

**3.1 We suggest regular physical exercise for the treatment of type 2 diabetes.**

*Strength of the recommendation: weak. Quality of evidence: low.*

**3.2. There is no evidence to prefer a threshold of 150 minutes per week for aerobic training in the treatment of type 2 diabetes.**

*Strength of the recommendation: weak. Quality of evidence: low.*

**3.3. We suggest combined (aerobic and resistance) training, rather than aerobic training alone, for the treatment of type 2 diabetes. **

4. **Educational therapy**

**4.1 We suggest structured educational therapy for the treatment of type 2 diabetes.**

*Strength of the recommendation: weak. Quality of evidence: very low.*

**4.2. We suggest grouped-based educational programs, rather than individual, for the treatment of type 2 diabetes.**

*Strength of the recommendation: weak. Quality of evidence: very low.*

5. **Pharmacological treatment**

**5.1 We recommend the use of metformin as first-line long-term treatment in patients with type 2 diabetes, without previous cardiovascular events. SGLT-2 inhibitors or GLP-1 receptor agonists are recommended as second-line treatments. Pioglitazone, DPP-4 inhibitors, acarbose and insulin should be considered as third-line treatments** (Figure 1).

*Strength of the recommendation: strong. Quality of evidence: moderate.*

**5.2.1. We recommend the use of metformin, SGLT-2 inhibitors or GLP-1 receptor agonists as first-line long-term treatment in patients with type 2 diabetes with previous cardiovascular events and without heart failure. DPP-4 inhibitors, pioglitazone, acarbose and insulin should be considered as second-line treatments** (Figure 1).

*Strength of the recommendation: strong. Quality of evidence: moderate.*

**5.2.2. We recommend the use of SGLT-2 inhibitors as first-line long-term treatment in patients with type 2 diabetes with previous heart failure. GLP-1 receptor agonists and metformin should be considered as second-line treatments. DPP-4 inhibitors, acarbose and insulin should be considered as third-line treatments** (Figure 1).

*Strength of the recommendation: strong. Quality of evidence: moderate.*

**5.3. We recommend the use of basal insulin analogues, instead of NPH, for all patients with type 2 diabetes needing treatment with basal insulin.**

*Strength of the recommendation: strong. Quality of evidence: very low.*

**5.4. We suggest the use of prandial insulin analogues for patients with type 2 diabetes needing treatment with prandial insulin.**

*Strength of the recommendation: weak. Quality of evidence: very low.*

**5.5. The routine use of continuous subcutaneous insulin infusion in inadequately controlled patients with type 2 diabetes is not recommended.**

*Strength of the recommendation: weak. Quality of evidence: very low.*

6. **Glycemic monitoring**

**6.1 We suggest to structure (with a pre-defined scheme of required tests) capillary blood glucose self-monitoring in the treatment of type 2 diabetes.**

*Strength of the recommendation: weak. Quality of evidence: very low.*

**6.2. We do not suggest a continuous glucose monitoring (continuous or on demand) rather than self-monitoring blood glucose in patients with type 2 diabetes on basal–bolus insulin therapy.**

*Strength of the recommendation: weak. Quality of evidence: very low.*

**1. THERAPEUTIC TARGETS**

**1.1 HbA1c target in patients treated with drugs inducing hypoglycemia**

Question: Which is the target HbA1c in patients with type 2 diabetes who are not treated with drugs capable of inducing hypoglycemia (insulin, sulfonylureas, glinides)?

**Table:** 

*Population*	People with type 2 diabetes treated with hypoglycemia-inducing drugs
*Intervention*	Intensified glucose control
*Comparison*	Standard glucose control
*Outcome*	Diabetic complications
*Setting*	Outpatient

**Relevant outcomes**

**Table:** 

Outcome	Relevance (1–9)	Critical
Microvascular complications	9	Yes
All-cause mortality	8	Yes
Severe hypoglycemia	8	Yes
Cardiovascular complications	7	Yes
Symptoms of diabetes	2	No

**RECOMMENDATION:**

**A target HbA1c between 49 mmol/mol (6.6%) and 58 mmol/mol (7.5%) is recommended for patients with type 2 diabetes treated with drugs capable of inducing hypoglycemia.**

*Strength of the recommendation: strong. Quality of evidence: low*

**Justification.** Several randomized trials show that the intensification of glucose control prevents long-term complications of diabetes, suggesting the need to reach and maintain HbA1c levels below 58 mmol/mol (7.5%). Lower targets (i.e., HbA1c < 48 mmol/mol or 6.5%) further reduce the risk of microvascular complications, but not of cardiovascular disease or mortality; however, a very strict glycemic control increases the risk of severe hypoglycemia, with an unfavorable risk/benefit ratio. For this reason, the most convenient HbA1c range for patients treated with drugs capable of inducing hypoglycemia is between 69 and 58 mmol/mol (6.6–7.5%). Higher targets can be considered for patients aged > 75 years or with reduced life expectancy because of comorbidities.

***Subgroup considerations.*** There are no available data from randomized trials on the safety and efficacy of intensification of glucose control in patients aged > 75 years; in addition, benefits of long-term glucose control are evident only after 2 years of treatment. This could motivate higher HbA1c targets in patients aged > 75 years or with reduced life expectancy because of comorbidities.

***Implementation.*** Specific programs for continuous medical education should be planned, to increase the awareness of healthcare professionals of the benefits of adequate glycemic control and the risks associated with very low HbA1c values in patients treated with hypoglycemia-inducing drugs.

***Assessment and monitoring.*** Adherence to this guideline can be assessed by estimating the proportion of patients at HbA1c target in existing databases.

**Assessment**

**Table:** 

**Problem** Is the problem a priority?		
**Judgment**	**Research evidence**	**Additional considerations**
Yes	The reduction of HbA1c levels in type 2 diabetes is associated with a lower risk of macro- and microvascular complications and mortality^12, 13^. However, there is a wide heterogeneity of results obtained with different strategies, in particular when using treatments associated or not with hypoglycemic risk^12−16^	
**Desirable Effects** How substantial are the desirable anticipated effects?		
**Judgment**	**Research evidence**	**Additional considerations**
Large	**Effects of HbA1c 49–58 mmol/mol (6.6–7.5%) on critical outcomes** ^17^ **:** MACE: −8%; Renal complications: −27% Ocular complications: −23% **Effects of HbA1c ≤ 48 mmol/mol (6.5%) on critical outcomes** ^17^ **:** Renal complications: −24% Ocular complications: −22% No significant effect on MACE, non-fatal myocardial infarction and stroke, all-cause and cardiovascular mortality	**Effect of intensification of treatment, irrespective of treatment strategies**^17^**:** (i.e., considering both drugs inducing and not inducing hypoglycemia)**:** MACE: −11%; Non-fatal myocardial infarction: −10% Non-fatal stroke: −11% Renal complications: −24% No significant effect on ocular complications, CV and all-cause mortality **Effect of intensification of treatment with drugs inducing hypoglycemia**^17^ (irrespective of glucose target): No significant effect on CV mortality MACE: −8%; Non-fatal MI: −15%; Non-fatal stroke: −15%; Ocular complications: −23%; Renal complications: −27% No evidence of heterogeneity in subgroup analyses No available trials enrolling patients aged over 75 years The observed benefits are evident only after at least 2 years of treatment
**Undesirable Effects** How substantial are the undesirable anticipated effects?		
**Judgment**	**Research evidence**	**Additional considerations**
Large	**Effects of HbA1c ≤ 58 mmol/mol (7.5%) on critical outcomes** ^17^ **:** (irrespective of glucose target): Severe hypoglycemia: OR: 2.72 [1.79, 4.13] **Effects of HbA1c ≤ 48 mmol/mol (6.5%) on critical outcomes** ^17^ **:** Severe hypoglycemia: OR: 2.62 [1.39, 4.97]	**Effect of intensification of treatment, irrespective of treatment strategies** (i.e., considering both drugs inducing and not inducing hypoglycemia)^17^**:** Severe hypoglycemia: 1.84 [1.20, 2.82] **Effect of intensification of treatment with drugs inducing hypoglycemia** (irrespective of glucose target): Severe hypoglycemia: 2.72 [1.79, 4.13] **Severe hypoglycemia was defined using the ADA criteria:** severe cognitive impairment requiring external assistance for recovery For UKPDS 33–34 Estimate, based on reported yearly incidence, assuming a recurrence rate of severe hypoglycemia
**Certainty of evidence** What is the overall certainty of the evidence of effects?		
**Judgment**	**Research evidence**	**Additional considerations**
Low	Moderate/low for all critical outcomes considered	
**Values** Is there important uncertainty about or variability in how much people value the main outcomes?		
**Judgment**	**Research evidence**	**Additional considerations**
No important uncertainty or variability	No evidence of variability or uncertainty Micro- and macrovascular complications and mortality are already considered among critical outcomes of the treatment of type 2 diabetes by scientific societies^4−6^	
**Balance of effects** Does the balance between desirable and undesirable effects favor the intervention or the comparison?		
**Judgment**	**Research evidence**	**Additional considerations**
Favors the intervention	The balance of effects of lowering HbA1c below 58 mmol/mol (7.5%) is favorable for the reduction of macro- and microvascular complications The balance of effects of lowering HbA1c below 48 mmol/mol (6.5%) is unfavorable because the risk of hypoglycemia outweighs the advantages of microvascular complications	
**Resources required** How large are the resource requirements (costs)?		
**Judgment**	**Research evidence**	**Additional considerations**
Varies	Small/moderate costs for intensification of therapy with some drugs (e.g., metformin), larger direct costs for insulin and newer agents ^18^	Results varied depending on drugs and contexts considered
**Certainty of evidence of required resources** What is the certainty of the evidence of resource requirements (costs)?		
**Judgment**	**Research evidence**	**Additional considerations**
High	Several good-quality studies explored this issue	
**Cost-effectiveness** Does the cost-effectiveness of the intervention favor the intervention or the comparison?		
**Judgment**	**Research evidence**	**Additional considerations**
Probably favors the intervention	The intensification of therapy is an effective means of preventing long-term complications of diabetes, thus determining a reduction of costs for the management of diabetic complications. Accordingly, intensification of therapy appears to be cost-effective at commonly accepted willingness to pay thresholds in the long-term horizon	
**Equity** What would be the impact on health equity?		
**Judgment**	**Research evidence**	**Additional considerations**
Probably increased	Epidemiological evidence suggests that different health professionals tend to adopt more conservative or more aggressive approaches toward diabetes treatment ^4–6^, depending on their background (e.g., specialists vs GPs) and geographical area. The adoption of evidence-based targets for HbA1c should improve health outcomes irrespective of the local organization of care and access to specialists	
**Acceptability** Is the intervention acceptable to key stakeholders?		
**Judgment**	**Research evidence**	**Additional considerations**
Probably yes	No specific evidence is available on this issue	
**Feasibility** Is the intervention feasible to implement?		
**Judgment**	**Research evidence**	**Additional considerations**
Yes	A relatively large proportion of patients with type 2 diabetes in Italy already falls within the recommended HbA1c targets ^4–6^	

**1.2 HbA1c target in patients not treated with drugs inducing hypoglycemia**

Question: Which is the target HbA1c in patients with type 2 diabetes who are not treated with drugs capable of inducing hypoglycemia (insulin, sulfonylureas, glinides)?

**Table:** 

*Population*	People with type 2 diabetes not treated with hypoglycemia-inducing drugs
*Intervention*	Intensified glucose control
*Comparison*	Standard glucose control
*Outcome*	Diabetic complications
*Setting*	Outpatient

**Relevant outcomes**

**Table:** 

Outcome	Relevance (1–9)	Critical
Microvascular complications	9	Yes
All-cause mortality	8	Yes
Cardiovascular complications	7	Yes
Severe hypoglycemia	2	No
Symptoms of diabetes	2	No

**RECOMMENDATION (1.2.1):**

**A target HbA1c below 53 mmol/mol (7%) is recommended for patients with type 2 diabetes not treated with drugs capable of inducing hypoglycemia.**

*Strength of the recommendation: strong. Quality of evidence: low.*

**Justification**. Several randomized trials show that the intensification of glucose control prevents long-term complications of diabetes, suggesting the need to reach and maintain HbA1c levels below 53 mmol/mol (7.0%). In particular, accurate glycemic control appears to reduce the risk of cardiovascular disease, with a variable cost/benefit ratio.

**Subgroup considerations**. There are no available data from randomized trials on the safety and efficacy of intensification of glucose control in patients aged > 75 years; in addition, benefits of long-term glucose control are evident only after 2 years of treatment. This could motivate higher HbA1c targets in patients aged > 75 years or with reduced life expectancy because of comorbidities.

**Implementation**. Specific programs for continuous medical education should be planned, to increase the awareness of healthcare professionals of the benefits of adequate glycemic control.

**Assessment and monitoring**. Adherence to this guideline can be assessed by estimating the proportion of patients at HbA1c target in existing databases^1,2^.

**RECOMMENDATION (1.2.2):**

**A target HbA1c of 48 mmol/mol (6.5%) or lower is suggested for patients with type 2 diabetes treated with drugs that are not capable of inducing hypoglycemia.**

*Strength of the recommendation: strong. Quality of evidence: low.*

**Justification**. No randomized trials assessed the effect of reaching and maintaining HbA1c ≤ 48 mmol/mol with drugs not capable of inducing hypoglycemia. Conversely, trials with hypoglycemia-inducing drugs show that the reduction of HbA1c below 48 mmol/mol prevents microvascular complications of diabetes. Pharmacoeconomic studies suggest that the achievement of this target, when obtained with drugs that do not induce hypoglycemia, reduces the need for hospitalization for diabetic complications, thus reducing overall health expenditure.

***Subgroup considerations.*** There are no available data from randomized trials on the safety and efficacy of intensification of glucose control in patients aged > 75 years; in addition, benefits of long-term glucose control are evident only after 2 years of treatment. This could motivate higher HbA1c targets in patients aged > 75 years or with reduced life expectancy because of comorbidities.

***Implementation.*** Specific programs for continuous medical education should be planned, to increase the awareness of healthcare professionals of the benefits of adequate glycemic control.

***Assessment and monitoring.*** Adherence to this guideline can be assessed by estimating the proportion of patients at HbA1c target in existing databases^19,20^.

**Assessment for HbA1c < 53 mmol/mol (7%)**

**Table:** 

**Problem** Is the problem a priority?		
**Judgment**	**Research evidence**	**Additional considerations**
Yes	The reduction of HbA1c levels in type 2 diabetes is associated with a lower risk of macro- and microvascular complications and mortality ^12, 13^. However, there is a wide heterogeneity of results obtained with different strategies, in particular when using treatments associated or not with hypoglycemic risk^12−16^	
**Desirable Effects** How substantial are the desirable anticipated effects?		
**Judgment**	**Research evidence**	**Additional considerations**
Large	**Effects of HbA1c 49–53 mmol/mol (6.6–7.0%) on critical outcomes** ^17^ **:** MACE: −22%; Non-fatal stroke: −23% No significant effect on non-fatal myocardial infarction and stroke, renal and ocular complications, and all-cause and cardiovascular mortality **Effects of HbA1c ≤ 54–58 mmol/mol (7.1–7.5%) on critical outcomes** ^17^ **:** MACE: −28%; Non-fatal stroke: −39% Renal complications: −31% No significant effect on non-fatal, all-cause and cardiovascular mortality. Increased risk for ocular complications (−75%) **Effects of HbA1c 59–64 mmol/mol (7.5–8.0%) on critical outcomes** ^17^ **:** All-cause mortality: −11%; Cardiovascular mortality: −12%; Renal complications: −31% No significant effect on MACE, non-fatal myocardial infarction, and stroke. No available data on ocular complications	**Effect of intensification of treatment, irrespective of treatment strategies**^17^**:** (i.e., considering both drugs inducing and not inducing hypoglycemia)**:** MACE: −11%; Non-fatal myocardial infarction: −10% Non-fatal stroke: − 11% Renal complications: − 24% No significant effect on ocular complications, CV and all-cause mortality **Effect of intensification of treatment with drugs not inducing hypoglycemia** (irrespective of glucose target) ^17^: No significant effect on ocular complications and non-fatal myocardial infarction MACE: − 15%; Non-fatal stroke: − 17%; Ocular complications: − 23%; All-cause and cardiovascular mortality: − 11%; Renal complications: − 30% Presence of heterogeneity for MACE and non-fatal ictus The observed benefits are evident only after at least 2 years of treatment
**Undesirable Effects** How substantial are the undesirable anticipated effects?		
**Judgment**	**Research evidence**	**Additional considerations**
Trivial	No increased risk of hypoglycemia^17^	**Effect of intensification of treatment, irrespective of treatment strategies** (i.e., considering both drugs inducing and not inducing hypoglycemia) ^17^**:** Severe hypoglycemia: 1.03 [0.88, 1.20 **Severe hypoglycemia was defined using the ADA criteria:** severe cognitive impairment requiring external assistance for recovery
**Certainty of evidence** What is the overall certainty of the evidence of effects?		
**Judgment**	**Research evidence**	**Additional considerations**
Low	High for MACE. Moderate for all-cause and cardiovascular mortality, and ocular complications. Low for renal complications	
**Values** Is there important uncertainty about or variability in how much people value the main outcomes?		
**Judgment**	**Research evidence**	**Additional considerations**
No important uncertainty or variability	No evidence of variability or uncertainty Micro- and macrovascular complications and mortality are already considered among critical outcomes of the treatment of type 2 diabetes by scientific societies^4−6^	
**Balance of effects** Does the balance between desirable and undesirable effects favor the intervention or the comparison?		
**Judgment**	**Research evidence**	**Additional considerations**
Favors the intervention	The balance of effects of lowering HbA1c below 53 mmol/mol (7.0%) is favorable for the reduction of macrovascular complications, with no additional risk of hypoglycemia	
**Resources required** How large are the resource requirements (costs)?		
**Judgment**	**Research evidence**	**Additional considerations**
Varies	Small/moderate costs for intensification of therapy with some drugs (e.g., metformin and pioglitazone), larger direct costs for insulin and newer agents ^18^	Results varied depending on drugs and contexts considered. Some drugs are generic or they will become soon, possibly reducing costs
**Certainty of evidence of required resources** What is the certainty of the evidence of resource requirements (costs)?		
**Judgment**	**Research evidence**	**Additional considerations**
High	Several good-quality studies explored this issue	
**Cost-effectiveness** Does the cost-effectiveness of the intervention favor the intervention or the comparison?		
**Judgment**	**Research evidence**	**Additional considerations**
Varies	The intensification of therapy is an effective means of preventing long-term complications of diabetes, thus determining a reduction of costs for the management of diabetic complications. Accordingly, intensification of therapy appears to be cost-effective at commonly accepted willingness to pay thresholds in the long-term horizon. Some newer agents despite their higher costs have shown some additional favorable effects on cerebro- and cardiovascular complications, thus increasing their cost-effectiveness	Newer agents, with higher direct costs, could become generic in the next months, thus increasing their cost-effectiveness
**Equity** What would be the impact on health equity?		
**Judgment**	**Research evidence**	**Additional considerations**
Probably increased	Epidemiological evidence suggests that different health professionals tend to adopt more conservative or more aggressive approaches toward diabetes treatment ^4–6^, depending on their background (e.g., specialists vs GPs) and geographical area. The adoption of evidence-based targets for HbA1c should improve health outcomes irrespective of the local organization of care and access to specialists	
**Acceptability** Is the intervention acceptable to key stakeholders?		
**Judgment**	**Research evidence**	**Additional considerations**
Probably yes	No specific evidence is available on this issue	
**Feasibility** Is the intervention feasible to implement?		
**Judgment**	**Research evidence**	**Additional considerations**
Yes	A relatively large proportion of patients with type 2 diabetes in Italy already falls within the recommended HbA1c targets^4−6^	

**Assessment for HbA1c < 48 mmol/mol (6.5%)**

**Table:** 

**Problem** Is the problem a priority?		
**Judgment**	**Research evidence**	**Additional considerations**
Yes	The reduction of HbA1c levels in type 2 diabetes is associated with a lower risk of macro- and microvascular complications and mortality ^12, 13^. However, there is a wide heterogeneity of results obtained with different strategies, particularly when using treatments associated or not with hypoglycemic risk^12−16^	
**Desirable Effects** How substantial are the desirable anticipated effects?		
**Judgment**	**Research evidence**	**Additional considerations**
Large	**Effects of HbA1c < 48 mmol/mol (6.5%) on critical outcomes** ^17^ **:** No available trial with a target lower than 48 mmol/mol (6.5%) Indirect evidence suggesting benefits on renal and ocular complications derive from trials with drugs inducing hypoglycemia and targets of HbA1c ≤ 48 mmol/mol (6.5%)	**Effect of intensification of treatment, irrespective of treatment strategies**^17^**:** (i.e., considering both drugs inducing and not inducing hypoglycemia)**:** MACE: − 11%; Non-fatal myocardial infarction: − 10% Non-fatal stroke: − 11% Renal complications: − 24% No significant effect on ocular complications, CV and all-cause mortality **Effect of intensification of treatment with drugs not inducing hypoglycemia** (irrespective of glucose target) ^17^: No significant effect on ocular complications and non-fatal myocardial infarction MACE: − 15%; Non-fatal stroke: − 17%; Ocular complications: − 23%; All-cause and cardiovascular mortality: − 11%; Renal complications: − 30% Presence of heterogeneity for MACE and non-fatal ictus The observed benefits are evident only after at least 2 years of treatment
**Undesirable Effects** How substantial are the undesirable anticipated effects?		
**Judgment**	**Research evidence**	**Additional considerations**
Trivial	No increased risk of hypoglycemia^17^	**Effect of intensification of treatment, irrespective of treatment strategies** (i.e., considering both drugs inducing and not inducing hypoglycemia) ^17^**:** Severe hypoglycemia: 1.03 [0.88, 1.20 **Severe hypoglycemia was defined using the ADA criteria:** severe cognitive impairment requiring external assistance for recovery
**Certainty of evidence** What is the overall certainty of the evidence of effects?		
**Judgment**	**Research evidence**	**Additional considerations**
Very low	Low for MACE and microvascular complications. Very low for the other critical outcomes	
**Values** Is there important uncertainty about or variability in how much people value the main outcomes?		
**Judgment**	**Research evidence**	**Additional considerations**
No important uncertainty or variability	No evidence of variability or uncertainty Micro- and macrovascular complications and mortality are already considered among critical outcomes of the treatment of type 2 diabetes by scientific societies^4−6, 20^	
**Balance of effects** Does the balance between desirable and undesirable effects favor the intervention or the comparison?		
**Judgment**	**Research evidence**	**Additional considerations**
Probably favors the intervention	The balance of effects of lowering HbA1c below 48 mmol/mol (6.5%) is unknown due to the lack of evidence. Indirect evidence suggests that targets < 48 mmol/mol obtained with drugs not inducing hypoglycemia could reduce the risk of microvascular complications	
**Resources required** How large are the resource requirements (costs)?		
**Judgment**	**Research evidence**	**Additional considerations**
Varies	Small/moderate costs for intensification of therapy with some drugs (e.g., metformin and pioglitazone), larger direct costs for insulin and newer agents^18^	Results varied depending on drugs and contexts considered. Some drugs are generic or they will become soon, possibly reducing costs
**Certainty of evidence of required resources** What is the certainty of the evidence of resource requirements (costs)?		
**Judgment**	**Research evidence**	**Additional considerations**
High	Several good-quality studies explored this issue	
**Cost-effectiveness** Does the cost-effectiveness of the intervention favor the intervention or the comparison?		
**Judgment**	**Research evidence**	**Additional considerations**
Varies	The intensification of therapy is an effective means of preventing long-term complications of diabetes, thus determining a reduction of costs for the management of diabetic complications. Accordingly, intensification of therapy appears to be cost-effective at commonly accepted willingness to pay thresholds in the long-term horizon. Some newer agents despite their higher costs have shown some additional favorable effects on cerebro- and cardiovascular complications, thus increasing their cost-effectiveness	Newer agents, with higher direct costs, could become generic in the next months, thus increasing their cost-effectiveness
**Equity** What would be the impact on health equity?		
**Judgment**	**Research evidence**	**Additional considerations**
Probably increased	Epidemiological evidence suggests that different health professionals tend to adopt more conservative or more aggressive approaches toward diabetes treatment^4−6^, depending on their background (e.g., specialists vs GPs) and geographical area. The adoption of evidence-based targets for HbA1c should improve health outcomes irrespective of the local organization of care and access to specialists	
**Acceptability** Is the intervention acceptable to key stakeholders?		
**Judgment**	**Research evidence**	**Additional considerations**
Probably yes	No specific evidence is available on this issue	
**Feasibility** Is the intervention feasible to implement?		
**Judgment**	**Research evidence**	**Additional considerations**
Yes	A relatively large proportion of patients with type 2 diabetes in Italy already falls within the recommended HbA1c targets^4−6^	

**2. NUTRITIONAL THERAPY**

**2.1 Structured Medical Nutrition Therapy vs unstructured nutritional advice**

Question: Is Medical Nutrition Therapy (MNT, composed of nutritional assessment, diagnosis, intervention and monitoring) preferable to simple nutritional recommendations for diabetes control in people with type 2 diabetes?

**Table:** 

*Population*	People with type 2 diabetes
*Intervention*	Structured Medical Nutrition Therapy
*Comparison*	Unstructured nutritional advice
*Outcome*	Glucose control
*Setting*	Outpatient

**Relevant outcomes**

**Table:** 

Outcome	Relevance (1–9)	Critical
Medium and long-term HbA1c	7	Yes
Body mass index	7	Yes
Treatment adherence	6	No
Patient’s preferences	6	No
Lipid profile	5	No
Hypoglycemia	3	No
Renal function	2	No

**RECOMMENDATION:**

**Structured Medical Nutrition Therapy is suggested for the treatment of type 2 diabetes**

*Strength of the recommendation: weak. Quality of evidence: low.*

***Justification.*** A small number of available trials, with methodological limitations and with relatively small sample size, show small but significant improvements in glycemic control and body weight with structured Medical Nutrition Therapy (MNT, composed of nutritional assessment, diagnosis, intervention and monitoring) when compared to unstructured nutritional advice. The low quality of evidence and the methodological biases of available studies limit the strength of this recommendation. Economic resources needed for implementation are negligible since unstructured nutritional advice is also time-consuming.

***Subgroup considerations.*** There are no available data from randomized trials on the safety and efficacy of MNT in patients aged > 75 years; in addition, patients with mental disorders and/or cognitive impairment could receive greater benefits from a traditional prescription of a diet, provided to the caregiver(s).

***Implementation.*** The awareness of healthcare professionals of the benefits of MNT could be increased by specific educational programs. The inclusion of MNT among indicators of the quality of care for diabetes could be of help in increasing adherence to this recommendation.

***Assessment and monitoring.*** The monitoring of this recommendation is problematic.

**Assessment**

**Table:** 

**Problem** Is the problem a priority?		
**Judgment**	**Research evidence**	**Additional considerations**
Yes	Nutritional recommendations are cornerstones of the management and therapy of type 2 diabetes Structured Medical Nutrition Therapy could provide long-term improvements in glycemic control and body weight Several trials have shown beneficial effects on HbA1c and body weight of structured Medical Nutrition Therapy (composed of nutritional assessment, diagnosis, intervention and monitoring) when compared to unstructured nutritional advice^21, 22^	
**Desirable Effects** How substantial are the desirable anticipated effects?		
**Judgment**	**Research evidence**	**Additional considerations**
Moderate	Improvement of^23^: HbA1c: − 0.45%; BMI: − 2 kg/m^2^	
**Undesirable Effects** How substantial are the undesirable anticipated effects?		
**Judgment**	**Research evidence**	**Additional considerations**
Trivial	This issue was not explored	
**Certainty of evidence** What is the overall certainty of the evidence of effects?		
**Judgment**	**Research evidence**	**Additional considerations**
Low	Low for both critical outcomes	
**Values** Is there important uncertainty about or variability in how much people value the main outcomes?		
**Judgment**	**Research evidence**	**Additional considerations**
No important uncertainty or variability	No evidence of variability or uncertainty HbA1c and BMI are already considered among critical outcomes of the treatment of type 2 diabetes by scientific societies^4−6^	
**Balance of effects** Does the balance between desirable and undesirable effects favor the intervention or the comparison?		
**Judgment**	**Research evidence**	**Additional considerations**
Probably favors the intervention	Small, but significant reduction of HbA1c and BMI, with no side effects	
**Resources required** How large are the resource requirements (costs)?		
**Judgment**	**Research evidence**	**Additional considerations**
Varies	The improvement of glycemic control and body weight reduction could theoretically determine cost saving in favor of the intervention, despite costs for personnel	It should be considered that unstructured nutritional advice is also time-consuming
**Certainty of evidence of required resources** What is the certainty of the evidence of resource requirements (costs)?		
**Judgment**	**Research evidence**	**Additional considerations**
Very low	Several low-quality studies explored this issue	
**Cost-effectiveness** Does the cost-effectiveness of the intervention favor the intervention or the comparison?		
**Judgment**	**Research evidence**	**Additional considerations**
Varies	Structured Medical Nutrition Therapy could be cost-effective. Economic resources needed for implementation are negligible since unstructured nutritional advice is also time-consuming	
**Equity** What would be the impact on health equity?		
**Judgment**	**Research evidence**	**Additional considerations**
Varies	No relevant differences in costs and accessibility, except for patients living far from the Outpatients clinic. This latter point could generate some equity problems	
**Acceptability** Is the intervention acceptable to key stakeholders?		
**Judgment**	**Research evidence**	**Additional considerations**
Probably yes	No specific evidence is available on this issue	
**Feasibility** Is the intervention feasible to implement?		
**Judgment**	**Research evidence**	**Additional considerations**
Yes	A relatively large proportion of patients with type 2 diabetes in Italy already received structured medical nutritional therapy^4−6^	Diabetes units have often the required resources to provide structured medical nutritional therapy (i.e., dietitians, nurses, physicians, etc.)

**2.2 Low carbohydrate vs balanced (Mediterranean) diet**

Question: Are low carbohydrate diets more effective than balanced (Mediterranean) diets for glucose control in people with type 2 diabetes?

**Table:** 

*Population*	People with type 2 diabetes
*Intervention*	Low carbohydrate diet
*Comparison*	Balanced (Mediterranean) diet
*Outcome*	Glucose control
*Setting*	Outpatient

**Relevant outcomes**

**Table:** 

Outcome	Relevance (1–9)	Critical
Medium and long-term HbA1c	7	Yes
Body mass index	7	Yes
Treatment adherence	6	No
Patient’s preferences	6	No
Lipid profile	5	No
Hypoglycemia	5	No
Renal function	5	No

**RECOMMENDATION:**

**We suggest a balanced (Mediterranean) diet, rather than a low-carbohydrate diet, for the treatment of type 2 diabetes.**

*Strength of the recommendation: weak. Quality of evidence: low.*

**Justification.** Few studies with methodological biases and a small number of included patients show small, but significant advantages on glycemic control of a balanced (Mediterranean) diet, when compared to a low-carbohydrate diet. The low quality of evidence and the methodological biases of available studies limit the strength of this recommendation. Economic resources needed for implementation are assumed as negligible, although no specific pharmacoeconomic studies were retrieved.

***Subgroup considerations.*** No data are available on the long-term renal safety of low-carbohydrate diets. Patients with renal impairment are usually excluded from clinical trials.

***Implementation.*** The awareness of healthcare professionals of the advantages of a balanced diet could be increased by specific educational programs.

***Assessment and monitoring.*** The monitoring of this recommendation is problematic.

***Research priorities*.** Further trials with good methodological quality comparing balanced and low-carbohydrate diets and assessing renal function among predefined outcomes are needed, to increase the strength of this recommendation.

**Assessment**

**Table:** 

**Problem** Is the problem a priority?		
**Judgment**	**Research evidence**	**Additional considerations**
Probably yes	Previous guidelines for type 2 diabetic patients recommended the Mediterranean diet for the treatment of diabetes. However, several studies showed some short-term beneficial effects of low-carbohydrate diets (ketogenic, Paleolithic, hyperproteic diets) on health outcomes, including the reduction of body weight in non-diabetic obese patients. Based on these studies, some physicians suggested these diets also to patients with diabetes to ameliorate their glycemic control^24, 25^. However, other studies suggested that the Mediterranean diet could have greater long-term effects^26^	
**Desirable Effects** How substantial are the desirable anticipated effects?		
**Judgment**	**Research evidence**	**Additional considerations**
Trivial	No between-group differences for HbA1c and body weight at 12 months^27^	
**Undesirable Effects** How substantial are the undesirable anticipated effects?		
**Judgment**	**Research evidence**	**Additional considerations**
Small	Small but statistically significant increase of HbA1c vs control diet (HbA1c: + 0.2%) at 24 months^27^	Only a few trials reported kidney function at the end of the study. This prevents the evaluation of the safety of low-carbohydrate diets (hyperproteic diets) on kidney function^27^
**Certainty of evidence** What is the overall certainty of the evidence of effects?		
**Judgment**	**Research evidence**	**Additional considerations**
Low	Low for both critical outcomes	
**Values** Is there important uncertainty about or variability in how much people value the main outcomes?		
**Judgment**	**Research evidence**	**Additional considerations**
No important uncertainty or variability	No evidence of variability or uncertainty HbA1c and BMI are already considered among critical outcomes of the treatment of type 2 diabetes by scientific societies^4−6^	
**Balance of effects** Does the balance between desirable and undesirable effects favor the intervention or the comparison?		
**Judgment**	**Research evidence**	**Additional considerations**
Probably favors the intervention	Small, but significant increase of HbA1c in favor of hypocaloric diet at 24 months	
**Resources required** How large are the resource requirements (costs)?		
**Judgment**	**Research evidence**	**Additional considerations**
Varies	No additional costs	Costs for protein-enriched food supplements could be higher than that for balanced diets
**Certainty of evidence of required resources** What is the certainty of the evidence of resource requirements (costs)?		
**Judgment**	**Research evidence**	**Additional considerations**
No included studies	No studies explored this issue	
**Cost-effectiveness** Does the cost-effectiveness of the intervention favor the intervention or the comparison?		
**Judgment**	**Research evidence**	**Additional considerations**
No included studies	No studies explored this issue	
**Equity** What would be the impact on health equity?		
**Judgment**	**Research evidence**	**Additional considerations**
Probably no impact	No relevant differences in costs and accessibility	
**Acceptability** Is the intervention acceptable to key stakeholders?		
**Judgment**	**Research evidence**	**Additional considerations**
Varies	The mean consumption of carbohydrates in Italy is considerably higher than that recommended in low-carbohydrates diets^28^	The acceptability of a low-carbohydrates diet could be problematic for patients with type 2 diabetes living in Italy due to the modifications imposed by the low-carbohydrates diets
**Feasibility** Is the intervention feasible to implement?		
**Judgment**	**Research evidence**	**Additional considerations**
Probably yes	No additional resources are required	

**3. PHYSICAL EXERCISE**

**3.1 Physical exercise and type 2 diabetes**

Question: Should physical exercise be recommended for diabetes control in patients with type 2 diabetes?

**Table:** 

*Population*	People with type 2 diabetes
*Intervention*	Physical exercise
*Comparison*	No intervention
*Outcome*	Glucose control, body weight and composition
*Setting*	Outpatient

**Relevant outcomes**

**Table:** 

Outcome	Relevance (1–9)	Critical
HbA1c	8	Yes
Body mass index	7	Yes
Fat mass	7	Yes
Patient’s preferences	6	No
Lipid profile	6	No
Hypoglycemia	6	No

**RECOMMENDATION:**

**We suggest regular physical exercise for the treatment of type 2 diabetes.**

*Strength of the recommendation: weak. Quality of evidence: moderate.*

***Justification.*** Several epidemiological studies showed beneficial effects of physical exercise on health outcomes, including the reduction of HbA1c and body weight, with no side effects and relevant costs, in type 2 diabetes^29^. The quality of available evidence is sufficient for drawing a recommendation, but some methodological flaws and the scarce number of patients included in the available studies downgrade the strength of this guideline.

***Subgroup considerations.*** There are no available data from randomized trials on the safety and efficacy of physical exercise in elderly patients.

***Implementation.*** The awareness of healthcare professionals of the benefits of physical exercise could be increased by specific educational programs. The inclusion of physical exercise among indicators of the quality of care for diabetes could be of help in increasing adherence to this recommendation.

***Assessment and monitoring.*** The monitoring of this recommendation is problematic.

**Assessment**

**Table:** 

**Problem** Is the problem a priority?		
**Judgment**	**Research evidence**	**Additional considerations**
Yes	Several national and international guidelines recommend physical exercise to ameliorate gluco-metabolic control in subjects with type 2 diabetes^4−6^. Several epidemiological studies showed beneficial effects of physical exercise on health outcomes, including the reduction of HbA1c, in type 2 diabetes^1^	
**Desirable Effects** How substantial are the desirable anticipated effects?		
**Judgment**	**Research evidence**	**Additional considerations**
Small	Improvement of^30^: HbA1c: − 0.3%; BMI: − 0.6 kg/m^2^; Fat mass: − 1.7%	
**Undesirable Effects** How substantial are the undesirable anticipated effects?		
**Judgment**	**Research evidence**	**Additional considerations**
Trivial	No relevant risk associated with physical exercise was detected in available RCTs^30^:	The risk of hypoglycemia should be always considered among patients treated with insulin and/or insulin secretagogues
**Certainty of evidence** What is the overall certainty of the evidence of effects?		
**Judgment**	**Research evidence**	**Additional considerations**
Very low	Moderate for HbA1c; Low for BMI; Very low for fat mass	
**Values** Is there important uncertainty about or variability in how much people value the main outcomes?		
**Judgment**	**Research evidence**	**Additional considerations**
No important uncertainty or variability	No evidence of variability or uncertainty HbA1c and BMI are already considered among critical outcomes of the treatment of type 2 diabetes by scientific societies^4−6^	
**Balance of effects** Does the balance between desirable and undesirable effects favor the intervention or the comparison?		
**Judgment**	**Research evidence**	**Additional considerations**
Probably favors the intervention	Small, but significant reduction of HbA1c, fat mass, and BMI, with no side effects	
**Resources required** How large are the resource requirements (costs)?		
**Judgment**	**Research evidence**	**Additional considerations**
Trivial	The recommendation of physical exercise does not require any additional costs^31^	It should be considered that some type of physical exercise (resistance exercise) could require some additional (not reimbursable) cost. However, many types of exercise are at very low costs
**Certainty of evidence of required resources** What is the certainty of the evidence of resource requirements (costs)?		
**Judgment**	**Research evidence**	**Additional considerations**
Very low	Several low-quality studies explored this issue ^31, 32^	
**Cost-effectiveness** Does the cost-effectiveness of the intervention favor the intervention or the comparison?		
**Judgment**	**Research evidence**	**Additional considerations**
Favors the interventions	The intervention appears cost-effective^31, 32^	
**Equity** What would be the impact on health equity?		
**Judgment**	**Research evidence**	**Additional considerations**
Varies	No specific evidence is available on this issue	No expected differences in costs and accessibility. However, the lack of dedicated public structures in some geographic areas could generate some equity problems
**Acceptability** Is the intervention acceptable to key stakeholders?		
**Judgment**	**Research evidence**	**Additional considerations**
Probably yes	No specific evidence is available on this issue	
**Feasibility** Is the intervention feasible to implement?		
**Judgment**	**Research evidence**	**Additional considerations**
Yes	This recommendation is already present in the principal national and international guidelines^4−6^	The recommendation of practicing physical exercise can be added during the routine visits

**3.2 Aerobic physical exercise and duration**

Question: Which is the minimum recommended duration of aerobic physical exercise for diabetes control in patients with type 2 diabetes?

**Table:** 

*Population*	People with type 2 diabetes
*Intervention*	Physical exercise > 150 min/week
*Comparison*	Physical exercise ≤ 150 min/week
*Outcome*	Glucose control, body weight and composition
*Setting*	Outpatient

**Relevant outcomes**

**Table:** 

Outcome	Relevance (1–9)	Critical	
HbA1c	8	Yes	
Body mass index	7	Yes	
Fat mass	7	Yes	
Patient’s preferences	6	No	
Lipid profile	6	No	
Hypoglycemia	6	No	

**RECOMMENDATION:**

**There is no evidence to prefer a threshold of 150 min per week for aerobic training in the treatment of type 2 diabetes.**

*Strength of the recommendation: weak. Quality of evidence: low.*

There are no studies directly comparing interventions with different goals for weekly exercise. The available evidence, derived from the indirect comparisons of trials comparing aerobic training of different duration with no exercise, is insufficient to detect either benefit or harms. The quality of available evidence is insufficient because of publication bias and methodological flaws.

***Subgroup considerations.*** None.

***Implementation.*** None.

***Assessment and monitoring.*** Not necessary.

**Assessment**

**Table:** 

**Problem** Is the problem a priority?		
**Judgment**	**Research evidence**	**Additional considerations**
Probably yes	In epidemiological studies, there is a relationship between the amount of aerobic exercise (at least 150 min/week) and health outcomes^33−35^. The identification of a minimum useful threshold of the duration of physical exercise needed for a therapeutic effect in type 2 diabetes is clinically relevant	
**Desirable Effects** How substantial are the desirable anticipated effects?		
**Judgment**	**Research evidence**	**Additional considerations**
Trivial	No differences in HbA1c, BMI, and fat mass^30^	
**Undesirable Effects** How substantial are the undesirable anticipated effects?		
**Judgment**	**Research evidence**	**Additional considerations**
Trivial	No relevant risk associated with physical exercise duration was detected in available RCTs^30^	
**Certainty of evidence** What is the overall certainty of the evidence of effects?		
**Judgment**	**Research evidence**	**Additional considerations**
Very low	Very low for all critical outcomes	
**Values** Is there important uncertainty about or variability in how much people value the main outcomes?		
**Judgment**	**Research evidence**	**Additional considerations**
No important uncertainty or variability	No evidence of variability or uncertainty HbA1c and BMI are already considered among critical outcomes of the treatment of type 2 diabetes by scientific societies^4−6^	
**Balance of effects** Does the balance between desirable and undesirable effects favor the intervention or the comparison?		
**Judgment**	**Research evidence**	**Additional considerations**
Does not favor either the intervention or the comparison	No between-group differences for any of the critical outcomes were considered	
**Resources required** How large are the resource requirements (costs)?		
**Judgment**	**Research evidence**	**Additional considerations**
Trivial	No specific evidence is available on this issue	
**Certainty of evidence of required resources** What is the certainty of the evidence of resource requirements (costs)?		
**Judgment**	**Research evidence**	**Additional considerations**
Very low	No specific evidence is available on this issue	
**Cost-effectiveness** Does the cost-effectiveness of the intervention favor the intervention or the comparison?		
**Judgment**	**Research evidence**	**Additional considerations**
Does not favor either the intervention or the comparison	No specific evidence is available on this issue	
**Equity** What would be the impact on health equity?		
**Judgment**	**Research evidence**	**Additional considerations**
Probably no impact	No expected differences in costs and accessibility	
**Acceptability** Is the intervention acceptable to key stakeholders?		
**Judgment**	**Research evidence**	**Additional considerations**
Probably yes	No specific evidence is available on this issue	
**Feasibility** Is the intervention feasible to implement?		
**Judgment**	**Research evidence**	**Additional considerations**
Yes	No additional costs or resources are required	

**3.3 Different modalities of physical exercise**

Question: Should combined aerobic/resistance training be preferred to aerobic training only for diabetes control in patients with type 2 diabetes?

**Table:** 

*Population*	People with type 2 diabetes
*Intervention*	Physical exercise
*Comparison*	Combined aerobic/resistance training
*Outcome*	Glucose control
*Setting*	Outpatient

**Relevant outcomes**

**Table:** 

Outcome	Relevance (1–9)	Critical	
HbA1c	7	Yes	
Body mass index	6	No	
Fat mass	6	No	
Patient’s adherence	6	No	
Hypoglycemia	3	No	
Lipid profile	2	No	

**RECOMMENDATION:**

**We suggest combined (aerobic and resistance) training, rather than aerobic training alone, for the treatment of type 2 diabetes.**

*Strength of the recommendation: weak. Quality of evidence: low.*

The preference for combined aerobic and resistance training was based on the greater reduction of HbA1c reported in available trials. The small between-group difference in HbA1c and the small sample size limit the strength of this recommendation. No issues of sustainability or equity were identified. The quality of available evidence is poor because of the limited sample size and of some methodological issues in clinical trials.

***Subgroup considerations.*** Some subpopulations of patients with type 2 diabetes (e.g., advanced age, heart failure, etc.) could benefit more from other modalities of physical exercise different from aerobic training.

***Implementation.*** The medical community should be made aware of the potential advantages of combined aerobic/anaerobic training through CME programs dedicated to non-pharmacological treatments of type 2 diabetes.

***Assessment and monitoring.*** The monitoring of adherence to guidelines on recommendations regarding non-pharmacological interventions and lifestyle behavior is problematic.

**Assessment**

**Table:** 

**Problem** Is the problem a priority?		
**Judgment**	**Research evidence**	**Additional considerations**
Probably yes	Aerobic exercise at least 3 days per week was recommended by most guidelines^4−6^. Resistance exercise alone or combined aerobic and resistance exercise was recommended only by a few guidelines^36, 37^. The identification of the best modality of physical exercise could be a relevant problem for the treatment of type 2 diabetes. Different types of exercise, which have differential effects on body composition, could theoretically determine different outcomes in diabetes control^29^	
**Desirable Effects** How substantial are the desirable anticipated effects?		
**Judgment**	**Research evidence**	**Additional considerations**
Small	Improvement of: HbA1c: − 0.2% (in favor of combined exercise)^30^	
**Undesirable Effects** How substantial are the undesirable anticipated effects?		
**Judgment**	**Research evidence**	**Additional considerations**
Trivial	No relevant risk associated with combined physical exercise was detected in available RCTs^30^	A post hoc analysis of the trials conducted for the present recommendation^30^ showed that combined exercise did not negatively affect blood pressure values at endpoint (systolic and diastolic blood pressure vs. aerobic exercise: −6.1 [−10.0, −2.3] mmHg and −2.8 [−6.3, 0.63] mmHg, respectively)
**Certainty of evidence** What is the overall certainty of the evidence of effects?		
**Judgment**	**Research evidence**	**Additional considerations**
Very low	Very low for HbA1c	
**Values** Is there important uncertainty about or variability in how much people value the main outcomes?		
**Judgment**	**Research evidence**	**Additional considerations**
No important uncertainty or variability	No evidence of variability or uncertainty HbA1c is already considered among critical outcomes of the treatment of type 2 diabetes by scientific societies^4−6^	
**Balance of effects** Does the balance between desirable and undesirable effects favor the intervention or the comparison?		
**Judgment**	**Research evidence**	**Additional considerations**
Probably favors the intervention	Small, but significant reduction of HbA1c	
**Resources required** How large are the resource requirements (costs)?		
**Judgment**	**Research evidence**	**Additional considerations**
Trivial	Similar overall expenditure between the two interventions, with a reported advantage on cost for QALY for combined training^31^	
**Certainty of evidence of required resources** What is the certainty of the evidence of resource requirements (costs)?		
**Judgment**	**Research evidence**	**Additional considerations**
Very low	No specific evidence is available on this issue^31^	
**Cost-effectiveness** Does the cost-effectiveness of the intervention favor the intervention or the comparison?		
**Judgment**	**Research evidence**	**Additional considerations**
Probably favors the intervention	Small, but significant improvement of HbA1c. Similar overall expenditure between the two interventions, with a reported advantage on cost for QALY for combined training^31^	
**Equity** What would be the impact on health equity?		
**Judgment**	**Research evidence**	**Additional considerations**
Probably no impact	No expected differences in costs and accessibility	
**Acceptability** Is the intervention acceptable to key stakeholders?		
**Judgment**	**Research evidence**	**Additional considerations**
Probably yes	No specific evidence is available on this issue	
**Feasibility** Is the intervention feasible to implement?		
**Judgment**	**Research evidence**	**Additional considerations**
Yes	No additional costs or resources are required	

**4. EDUCATIONAL THERAPY**

**4.1 Structured educational therapy**

Question: Should structured educational therapy be preferable in comparison with generic advice for diabetes control in patients with type 2 diabetes?

**Table:** 

*Population*	People with type 2 diabetes
*Intervention*	Structured educational therapy
*Comparison*	Non-structured educational therapy
*Outcome*	HbA1c, hypoglycemia, short/medium-term adherence, quality of life
*Setting*	Outpatient

**Relevant outcomes**

**Table:** 

Outcome	Relevance (1–9)	Critical	
HbA1c	8	Yes	
Medium/long-term patient’s adherence	7	Yes	
Hypoglycemia	7	Yes	
Quality of life	7	Yes	
Body mass index	6	No	

**RECOMMENDATION:**

**We suggest structured educational therapy for the treatment of type 2 diabetes.**

*Strength of the recommendation: weak. Quality of evidence: very low.*

***Justification.*** The preference for grouped-based educational programs is based on the possible better glycemic control, weight loss, quality of life and reduced costs. The quality of available evidence is poor because of the limited sample size and of some methodological issues in clinical trials, thus reducing the strength of this recommendation.

***Subgroup considerations.*** Few available data on elderly patients do not allow to assess the efficacy of the structured educational therapy in the advanced decades. Patients with psychiatric disorders or cognitive impairment could benefit more from traditional education often managed by caregivers.

***Implementation.*** The medical community should be made aware of the potential advantages of structured educational therapy through CME programs dedicated to non-pharmacological treatments of type 2 diabetes.

***Assessment and monitoring***. The monitoring of adherence to guidelines on recommendations regarding non-pharmacological interventions and lifestyle behavior is problematic.

**Assessment**

**Table:** 

**Problem** Is the problem a priority?		
**Judgment**	**Research evidence**	**Additional considerations**
Yes	Educational therapy is usually part of the clinical management of type 2 diabetes and is recommended by the most important guidelines^4−6^. The adoption of structured educational programs could ameliorate long-term glucose control Several studies showed beneficial effects of structured educational therapy on health outcomes, including the reduction of HbA1c and body weight in type 2 diabetes^38−40^	
**Desirable Effects** How substantial are the desirable anticipated effects?		
**Judgment**	**Research evidence**	**Additional considerations**
Moderate	**Effects of structured educational therapy** ^41^ **:** HbA1c: − 0.35% Quality of life: no effect on generic questionnaires; improvement of diabetes-specific QoL	
**Undesirable Effects** How substantial are the undesirable anticipated effects?		
**Judgment**	**Research evidence**	**Additional considerations**
Trivial	No expected differences	
**Certainty of evidence** What is the overall certainty of the evidence of effects?		
**Judgment**	**Research evidence**	**Additional considerations**
Very low	Very low for QoL; Low for all the other clinical outcomes	
**Values** Is there important uncertainty about or variability in how much people value the main outcomes?		
**Judgment**	**Research evidence**	**Additional considerations**
Probably relevant	No evidence of variability or uncertainty HbA1c is already considered among critical outcomes of the treatment of type 2 diabetes by scientific societies^4−6^. However, it is conceivable that educational therapy can have different effects based on patient's characteristics (e.g., duration of diabetes; type of therapy—injectable vs. non-injectable drugs—cognitive status, etc.)	
**Balance of effects** Does the balance between desirable and undesirable effects favor the intervention or the comparison?		
**Judgment**	**Research evidence**	**Additional considerations**
Probably favors the intervention	Small, but significant reduction of HbA1c and favorable effects on QoL, with no reported side effects	
**Resources required** How large are the resource requirements (costs)?		
**Judgment**	**Research evidence**	**Additional considerations**
Trivial	Structured educational therapy could be cost-effective due to the reduction of HbA1c and amelioration of QoL. These favorable effects could contribute to the reduction of costs for long-term complications despite the increased direct costs for the implementation of educational programs	It should be considered that unstructured educational advice is also time-consuming
**Certainty of evidence of required resources** What is the certainty of the evidence of resource requirements (costs)?		
**Judgment**	**Research evidence**	**Additional considerations**
Moderate	No specific evidence is available on this issue	
**Cost-effectiveness** Does the cost-effectiveness of the intervention favor the intervention or the comparison?		
**Judgment**	**Research evidence**	**Additional considerations**
Probably favors the intervention	Despite high heterogeneity, the structured educational therapy could be cost-effective due to limited additional costs to be implemented	
**Equity** What would be the impact on health equity?		
**Judgment**	**Research evidence**	**Additional considerations**
Varies	No expected differences in costs and accessibility	However, the lack of dedicated public structures in some geographic areas could generate some equity problems
**Acceptability** Is the intervention acceptable to key stakeholders?		
**Judgment**	**Research evidence**	**Additional considerations**
Probably yes	No specific evidence is available on this issue	
**Feasibility** Is the intervention feasible to implement?		
**Judgment**	**Research evidence**	**Additional considerations**
Yes	A relatively large proportion of patients with type 2 diabetes in Italy already received structured educational therapy^19, 20^	Diabetes units services have often the required resources to provide structured educational therapy (i.e., dietitians, nurses, physicians, etc.)

**4.2 Group- and individual-based educational therapy**

Question: Should group-based educational therapy be preferable in comparison with individual therapy for diabetes control in patients with type 2 diabetes?

**Table:** 

*Population*	People with type 2 diabetes
*Intervention*	Group-based educational therapy
*Comparison*	Individual-based educational therapy
*Outcome*	HbA1c, short/medium-term adherence, quality of life
*Setting*	Outpatient

**Relevant outcomes**

**Table:** 

Outcome	Relevance (1–9)	Critical
HbA1c	8	Yes
Medium/long-term patient’s adherence	7	Yes
Quality of life	7	Yes
Hypoglycemia	6	No
Body mass index	6	No

**RECOMMENDATION:**

**We suggest grouped-based educational programs, rather than individual, for the treatment of type 2 diabetes.**

*Strength of the recommendation: weak. Quality of evidence: very low.*

***Justification.*** The preference for grouped-based educational programs is based on the possible better quality of life and reduced costs. There is no effect on HbA1c, thus limiting the strength of this recommendation.

***Subgroup considerations.*** The possibility that some subgroup of patients can have some advantages on glucose control cannot be completely ruled out. Group-based therapy could determine better glycemic control in programs with longer duration and in non-insulin-treated patients with lower baseline HbA1c levels. Conversely, available clinical trials do not include very old patients, those with cognitive impairment and those with major psychiatric conditions.

***Implementation.*** The medical community should be made aware of the potential advantages of a macronutrient-balanced diet through CME programs dedicated to non-pharmacological treatments of type 2 diabetes.

***Assessment and monitoring***. The development of group education programs in Diabetes Outpatient Clinics could be monitored through the analysis of administrative data on performed activities.

**Assessment**

**Table:** 

**Problem** Is the problem a priority?		
**Judgment**	**Research evidence**	**Additional considerations**
Yes	Group-based education for individuals with type 2 diabetes may be more cost-effective and efficient than individual education, due to the reduced time and funding required The potential advantages of group-based education interventions over individual visits include a) time for the provision of more detailed information, b) decreased time demands on health workers, c) easier involvement of families and caregivers and d) facilitation of discussions and support from others facing the same challenges^42, 43^	
**Desirable Effects** How substantial are the desirable anticipated effects?		
**Judgment**	**Research evidence**	**Additional considerations**
Moderate	**Effects of group-based education**: No between-group difference in: HbA1c: and patients’ adherence Quality of life: improvement of diabetes-specific QoL (*Diabetes quality of life (DQOL)*: − 24.4[− 42.9;− 5.8])	No insulin-treated patients, with a longer duration of diabetes, higher baseline mean age and lower baseline mean HbA1c levels were more likely to benefit group-based programs (i.e., greater efficacy in reducing HbA1c) particularly in trials with longer duration
**Undesirable Effects** How substantial are the undesirable anticipated effects?		
**Judgment**	**Research evidence**	**Additional considerations**
Trivial	Not explored. No expected differences in side effects	
**Certainty of evidence** What is the overall certainty of the evidence of effects?		
**Judgment**	**Research evidence**	**Additional considerations**
Very low	Low for HbA1c; Very low for all the other clinical outcomes	
**Values** Is there important uncertainty about or variability in how much people value the main outcomes?		
**Judgment**	**Research evidence**	**Additional considerations**
No important uncertainty or variability	No evidence of variability or uncertainty HbA1c and QoL are already considered among critical outcomes of the treatment of type 2 diabetes by scientific societies^4−6^	
**Balance of effects** Does the balance between desirable and undesirable effects favor the intervention or the comparison?		
**Judgment**	**Research evidence**	**Additional considerations**
Probably favors the intervention	Possible favorable effects on QoL	Few trials report data on QoL^42, 44–46^
**Resources required** How large are the resource requirements (costs)?		
**Judgment**	**Research evidence**	**Additional considerations**
Moderate savings	Possibly lower costs	Variability related to the type of intervention
**Certainty of evidence of required resources** What is the certainty of the evidence of resource requirements (costs)?		
**Judgment**	**Research evidence**	**Additional considerations**
Very low	Few specific low-quality evidence is available on this issue	
**Cost-effectiveness** Does the cost-effectiveness of the intervention favor the intervention or the comparison?		
**Judgment**	**Research evidence**	**Additional considerations**
Probably favors the intervention	The intervention could be cost-effective	
**Equity** What would be the impact on health equity?		
**Judgment**	**Research evidence**	**Additional considerations**
Varies	No expected differences in costs and accessibility	
**Acceptability** Is the intervention acceptable to key stakeholders?		
**Judgment**	**Research evidence**	**Additional considerations**
Probably yes	No specific evidence is available on this issue	
**Feasibility** Is the intervention feasible to implement?		
**Judgment**	**Research evidence**	**Additional considerations**
Probably yes	No additional resources are required	

**5. PHARMACOLOGICAL THERAPY**

**5.1 Glucose-lowering therapy in patients with type 2 diabetes and no previous cardiovascular events**

Which glucose-lowering agents should be considered as first-, second- and third-line therapy for glycemic control in patients with type 2 diabetes and no previous cardiovascular events?

**Table:** 

*Population*	People with type 2 diabetes
*Intervention*	Glucose-lowering therapy
*Comparison*	Glucose-lowering therapy
*Outcome*	HbA1c, hypoglycemia, medium/long-term adherence, mortality; major cardiovascular events
*Setting*	Outpatient

**Relevant outcomes**

**Table:** 

Outcome	Relevance (1–9)	Critical
Hypoglycemia	9	Yes
Medium/long-term HbA1c	8	Yes
Quality of life	8	Yes
Major cardiovascular events	7	Yes
Body mass index	7	Yes
Renal function	6	No
Albuminuria	6	No
Hospitalization for heart failure	4	No
Short-term HbA1c	3	No
Genito-urinary infection	3	No
Ketosis	2	No

**RECOMMENDATION:**

**We recommend the use of metformin as a first-line long-term treatment in patients with type 2 diabetes without previous cardiovascular events. SGLT-2 inhibitors or GLP-1 receptor agonists are recommended as second-line treatments. Pioglitazone, DPP-4 inhibitors, acarbose and insulin should be considered as third-line treatments.**

*Strength of the recommendation: strong. Quality of evidence: low.*

**Justification.** A major body of evidence from randomized controlled trials supports the use of metformin, SGLT-2 inhibitors, or GLP-1 receptor agonists as first-line treatment in patients with type 2 diabetes due to relevant efficacy in reducing HbA1c without increasing the risk of hypoglycemia and less risk of MACE and all-cause mortality. Moreover, GLP-1 receptor agonists and SGLT-2 inhibitors also have beneficial effects on body weight. Insulin secretagogues have shown a lower efficacy in reducing HbA1c with a higher risk of hypoglycemia in comparison with metformin; in addition, a higher mortality rate was observed in comparison with other glucose-lowering agents/placebo, and therefore, their use should be avoided for the treatment of type 2 diabetes. The quality of available evidence is generally satisfactory. Several good-quality pharmacoeconomic studies showed that metformin has the lowest direct costs in comparison with other classes of glucose-lowering agents which have similar clinical effects.

***Subgroup considerations.*** This recommendation provides more than one option for both second- and third-line therapy. The choice among available options can be affected by patients' characteristics such as age, renal failure, body weight, duration of diabetes, comorbid conditions, diabetic complications, etc., or by clinical conditions (e.g., high degree of hyperglycemia) based on clinicians' Judgment.

***Implementation.*** Sulfonylureas should not be added to ongoing therapy; existing treatments with sulfonylureas should be progressively deprescribed or substitutes with other therapies irrespective of glycemic control.

The whole medical community should be made aware of this recommendation to homogenize the therapy for type 2 diabetes in line with evidence-based medicine. Continuous medical education programs are needed to implement the knowledge of physicians in this respect.

***Assessment and monitoring***. The monitoring of adherence to guidelines on the pharmacological treatment of type 2 diabetes can be implemented through the consultation of existing databases^7,8^.

**Assessment**

**Table:** 

**Problem** Is the problem a priority?		
**Judgment**	**Research evidence**	**Additional considerations**
Yes	Different guidelines propose different algorithms for the pharmacological treatment of type 2 diabetes. Many guidelines recommend metformin as first-line agents^4−6^, but others prefer other agents in the majority of patients^7^. Recommendations on second- and third-line therapy are also heterogeneous^4−7^ The preference for a drug over another depends on its safety and tolerability, as well as its efficacy. Some side effects (e.g., weight gain, hypoglycemia and gastrointestinal effects) are common with some glucose-lowering drugs. Those adverse effects, together with the complexity and potential burdens of therapy, may affect patients’ quality of life. In addition, several drugs have been shown renal and cardiovascular, and/or nefro-protective effects. All those factors should be considered when selecting a drug, or a combination of drugs, for the treatment of an individual patient	
**Desirable Effects** How substantial are the desirable anticipated effects?		
**Judgment**	**Research evidence**	**Additional considerations**
Varies	**Effects of different classes of drugs, as reported in direct comparisons**^47^ (only statistical significant results are reported): *52-week HbA1c: compared to metformin* GLP-1 RA: −0.2% Acarbose: + 0.4% *104-week HbA1c: compared to metformin* SGLT-2i: −0.2% Sulfonylureas: + 0.1% Insulin: + 0.4% **Overall effects of different classes on MACE:** Metformin: −48%^48^; GLP-1 RA: −11%^49^; SGLT-2i: −11%^50^ **Overall effects of different classes on all-cause mortality:** GLP-1 RA: −11%^49^; SGLT-2i: −14%^50^; Sulfonylureas: + 11%^51^. Despite the increased risk of mortality did not reach statistical significance in any of the trials considered, the overall mortality (combining all the trials using a meta-analytical approach) for sulfonylureas was higher in comparison with placebo/other classes **Quality of life** GLP-1RA are associated with improved quality of life in comparison with DPP4 inhibitors or insulin^49^	The effects on MACE and all-cause mortality derive from RCTs performed on patients with previous cardiovascular events
**Undesirable Effects** How substantial are the undesirable anticipated effects?		
**Judgment**	**Research evidence**	**Additional considerations**
Varies	Severe hypoglycemia: Sulphonylureas increase the risk of hypoglycemia (OR: 3.7) in comparison with metformin^47^	Metformin: gastrointestinal side effects; rare cases of lactic acidosis Alpha-glucosidase inhibitors: gastrointestinal side effects Sulfonylureas: weight gain; hypoglycemia Pioglitazone: fluid retention; weight gain; heart failure; bone fracture DPP-4 inhibitors: suspected pancreatitis; rare cases of pemphigoid GLP-1RA: gastrointestinal side effects; cholelithiasis; pancreatitis SGLT-2 inhibitors: genito-urinary infections; rare ketoacidosis Insulin: hypoglycemia and weight gain^51^
**Certainty of evidence** What is the overall certainty of the evidence of effects?		
**Judgment**	**Research evidence**	**Additional considerations**
Low	Moderate for MACE (pioglitazone and sulfonylureas); Low for all the other clinical outcomes	
**Values** Is there important uncertainty about or variability in how much people value the main outcomes?		
**Judgment**	**Research evidence**	**Additional considerations**
No important uncertainty or variability	No evidence of variability or uncertainty HbA1c, body weight, severe hypoglycemia, macrovascular complications and mortality are already considered among critical outcomes of the treatment of type 2 diabetes by scientific societies^4−6^	
**Balance of effects** Does the balance between desirable and undesirable effects favor the intervention or the comparison?		
**Judgment**	**Research evidence**	**Additional considerations**
Varies	The balance of effects favor metformin, GLP1 RA, and SGLT2i over other classes of drugs, whereas it is unfavorable for sulfonylureas	
**Resources required** How large are the resource requirements (costs)?		
**Judgment**	**Research evidence**	**Additional considerations**
Varies	Low for metformin, pioglitazone, sulfonylureas, acarbose Moderate for other classes, higher for GLP1RA and insulin	Some bioequivalent molecules could reduce direct costs for the most expensive approaches (i.e., insulin and GLP1RA)
**Certainty of evidence of required resources** What is the certainty of the evidence of resource requirements (costs)?		
**Judgment**	**Research evidence**	**Additional considerations**
High	Several good-quality studies explored this issue	
**Cost-effectiveness** Does the cost-effectiveness of the intervention favor the intervention or the comparison?		
**Judgment**	**Research evidence**	**Additional considerations**
Varies	The cost-effective evaluation depends on the form of the drug used	
**Equity** What would be the impact on health equity?		
**Judgment**	**Research evidence**	**Additional considerations**
Probably no impact	Drugs recommended in the present guideline are already considered as first-and second-line treatment for patients without previous cardiovascular events in the principal guidelines^4−6, 52^	
**Acceptability** Is the intervention acceptable to key stakeholders?		
**Judgment**	**Research evidence**	**Additional considerations**
Probably yes	No specific evidence is available on this issue	
**Feasibility** Is the intervention feasible to implement?		
**Judgment**	**Research evidence**	**Additional considerations**
Probably yes	A large part of patients with type 2 diabetes in Italy is already treated with metformin, whereas GLP-1 RA and SGLT-2i are still relatively underutilized and sulfonylureas still prescribed^19, 20^	

**5.2 Glucose-lowering therapy in patients with type 2 diabetes and previous cardiovascular events with or without heart failure**

**5.2.1 Question #1**

Which glucose-lowering agents should be considered as first-, second- and third-line therapy for glycemic control in patients with type 2 diabetes and previous cardiovascular events and without heart failure?

**Table:** 

*Population*	People with type 2 diabetes
*Intervention*	Glucose-lowering therapy
*Comparison*	Glucose-lowering therapy
*Outcome*	HbA1c, hypoglycemia, quality of life, mortality; major cardiovascular events; hospitalization for heart failure
*Setting*	Outpatient

**Relevant outcomes**

**Table:** 

Outcome	Relevance (1–9)	Critical
Major cardiovascular events	9	Yes
Hospitalization for heart failure	8	Yes
Hypoglycemia	8	Yes
Medium/long-term HbA1c	7	Yes
Quality of life	7	Yes
Body mass index	5	No
Renal function	6	No
Albuminuria	4	No
Short-term HbA1c	3	No
Genito-urinary infection	3	No
Ketosis	3	No

**RECOMMENDATION:**

**We recommend the use of metformin, SGLT-2 inhibitors, or GLP-1 receptor agonists as first-line long-term treatment in patients with type 2 diabetes with previous cardiovascular events and without heart failure. DPP-4 inhibitors, pioglitazone, acarbose, and insulin should be considered as second-line treatments.**

*Strength of the recommendation: strong. Quality of evidence: moderate.*

**Justification.** A major body of evidence from randomized controlled trials supports the use of metformin, SGLT-2 inhibitors, or GLP-1 receptor agonists as first-line treatment in patients with type 2 diabetes due to relevant efficacy in reducing HbA1c without increasing the risk of hypoglycemia and less risk of MACE and all-cause mortality. In particular, SGLT-2 inhibitors in comparison with metformin and GLP-1 receptor agonists, have favorable effects on the risk of hospitalization for heart failure. Moreover, GLP-1 receptor agonists and SGLT-2 inhibitors also have beneficial effects on body weight. Insulin secretagogues have shown a lower efficacy in reducing HbA1c with a higher risk of hypoglycemia in comparison with metformin; in addition, a higher mortality rate was observed in comparison with other glucose-lowering agents/placebo, and therefore, their use should be avoided for the treatment of type 2 diabetes. The quality of available evidence is generally satisfactory. Several good-quality pharmacoeconomic studies showed that metformin has the lowest direct costs in comparison with other classes of glucose-lowering agents; moreover, metformin and SGLT-2 inhibitors and, to a lesser extent, GLP-1 receptor agonists have a good cost-effective ratio.

***Subgroup considerations.*** This recommendation provides more than one option for both second- and third-line therapy. The choice among available options can be affected by patients' characteristics such as age, renal failure, body weight, duration of diabetes, comorbid conditions, diabetic complications, etc., or by clinical conditions (e.g., high degree of hyperglycemia) based on clinicians' Judgment.

***Implementation.*** Sulfonylureas should not be added to ongoing therapy; existing treatments with sulfonylureas should be progressively deprescribed or substitutes with other therapies irrespective of glycemic control. The whole medical community should be made aware of this recommendation to homogenize the therapy for type 2 diabetes in line with evidence-based medicine. Continuous medical education programs are needed to implement the knowledge of physicians in this respect.

***Assessment and monitoring***. The monitoring of adherence to guidelines on the pharmacological treatment of type 2 diabetes can be implemented through the consultation of existing databases.

**5.2.2. Question #2**

Which glucose-lowering agents should be considered as first-, second- and third-line therapy for glycemic control in patients with type 2 diabetes and previous heart failure?

**Table:** 

*Population*	People with type 2 diabetes
*Intervention*	Glucose-lowering therapy
*Comparison*	Glucose-lowering therapy
*Outcome*	HbA1c, hypoglycemia, quality of life; mortality; major cardiovascular events; and hospitalization for heart failure
*Setting*	Outpatient

**Relevant outcomes**

**Table:** 

Outcome	Relevance (1–9)	Critical
Hospitalization for heart failure	9	Yes
Quality of life	8	Yes
Major cardiovascular events	7	Yes
Hypoglycemia	7	Yes
Medium/long-term HbA1c	7	Yes
Renal function	5	No
Body mass index	4	No
Albuminuria	3	No
Short-term HbA1c	3	No
Ketosis	3	No
Genito-urinary infection	2	No

**RECOMMENDATION:**

**We recommend the use of SGLT-2 inhibitors as first-line long-term treatment in patients with type 2 diabetes with previous heart failure. GLP-1 receptor agonists and metformin should be considered as second-line treatments. DPP-4 inhibitors, acarbose and insulin should be considered as third-line treatments.**

*Strength of the recommendation: strong. Quality of evidence: moderate.*

**Justification.** A major body of evidence from randomized controlled trials supports the use of metformin, SGLT-2 inhibitors, or GLP-1 receptor agonists as first-line treatment in patients with type 2 diabetes due to relevant efficacy in reducing HbA1c without increasing the risk of hypoglycemia and less risk of MACE and all-cause mortality. In particular, SGLT-2 inhibitors in comparison with metformin and GLP-1 receptor agonists, have favorable effects on the risk of hospitalization for heart failure. Moreover, GLP-1 receptor agonists and SGLT-2 inhibitors also have beneficial effects on body weight. Insulin secretagogues have shown a lower efficacy in reducing HbA1c with a higher risk of hypoglycemia in comparison with metformin; in addition, a higher mortality rate was observed in comparison with other glucose-lowering agents/placebo, and therefore, their use should be avoided for the treatment of type 2 diabetes. The quality of available evidence is generally satisfactory. Several good-quality pharmacoeconomic studies showed that metformin has the lowest direct costs in comparison with other classes of glucose-lowering agents; moreover, metformin and SGLT-2 inhibitors and, to a lesser extent, GLP-1 receptor agonists have a good cost-effective ratio.

***Subgroup considerations.*** This recommendation provides more than one option for both second- and third-line therapy. The choice among available options can be affected by patients' characteristics such as age, renal failure, body weight, duration of diabetes, comorbid conditions, diabetic complications, etc., or by clinical conditions (e.g., high degree of hyperglycemia) based on clinicians' Judgment. Metformin can be used only in patients with NYHA < III. Saxagliptin should be avoided due to the high risk of hospitalization for heart failure.

***Implementation.*** Sulfonylureas should not be added to ongoing therapy; existing treatments with sulfonylureas should be progressively deprescribed or substitutes with other therapies irrespective of glycemic control. The whole medical community should be made aware of this recommendation to homogenize the therapy for type 2 diabetes in line with evidence-based medicine. Continuous medical education programs are needed to implement the knowledge of physicians with this respect.

***Assessment and monitoring***. The monitoring of adherence to guidelines on the pharmacological treatment of type 2 diabetes can be implemented through the consultation of existing databases.

**Assessment (both for questions #1 and #2)**

**Table:** 

**Problem** Is the problem a priority?		
**Judgment**	**Research evidence**	**Additional considerations**
Yes	Specific recommendations for patients with prior cardiovascular events are provided by some guidelines^4−6, 52^. The absolute risk of cardiovascular events and all-cause mortality is particularly increased in patients with type 2 diabetes and established cardiovascular disease. The risk reduction observed with some classes of drugs for diabetes could therefore produce very relevant benefits in this subset of patients with diabetes The availability of data on specific effects of some classes of drugs on the incidence of hospital admissions for heart failure suggests considering separately patients with previous cardiovascular events and known heart failure	
**Desirable Effects** How substantial are the desirable anticipated effects?		
**Judgment**	**Research evidence**	**Additional considerations**
Varies	**Effects of different classes of drugs, as reported in direct comparisons**^47^ (only statistical significant results are reported): *52-week HbA1c: compared to metformin* GLP-1 RA: −0.2% Acarbose: + 0.4% *104-week HbA1c: compared to metformin* SGLT-2i: −0.2% Sulfonylureas: + 0.1% Insulin: + 0.4% **Overall effects of different classes on MACE:** Metformin: − 48%^48^; GLP-1 RA: − 11%^49^; SGLT-2i: − 11%^50^ **Overall effects of different classes on hospitalization for heart failure** SGLT-2i: − 30% **Overall effects of different classes on all-cause mortality:** GLP-1 RA: − 11%^49^; SGLT-2i: − 14%^50^; Sulfonylureas: + 11%^51^ **Quality of life** GLP-1RA is associated with improved quality of life in comparison with DPP4 inhibitors or insulin^50^	MACE: no trial was found for alpha-glucosidase inhibitors For metformin, a sensitivity post hoc analysis including all RCT > 52 weeks, irrespective of the inclusion of major cardiovascular events within the principal endpoint or as a pre-defined secondary endpoint with formal adjudication of events, was performed confirming the reduction of the risk of MACE (−43%)^48^
**Undesirable Effects** How substantial are the undesirable anticipated effects?		
**Judgment**	**Research evidence**	**Additional considerations**
Varies	Severe hypoglycemia: Sulphonylureas increase the risk of hypoglycemia (OR: 3.7) in comparison with metformin^47^	Metformin: gastrointestinal side effects; rare cases of lactic acidosis Alpha-glucosidase inhibitors: gastrointestinal side effects Sulfonylureas: weight gain; hypoglycemia Pioglitazone: fluid retention; weight gain; heart failure; bone fracture DPP-4 inhibitors: suspected pancreatitis; rare cases of pemphigoid GLP-1RA: gastrointestinal side effects; cholelithiasis; pancreatitis SGLT-2 inhibitors: genito-urinary infections; rare ketoacidosis Insulin: hypoglycemia and weight gain^51^
**Certainty of evidence** What is the overall certainty of the evidence of effects?		
**Judgment**	**Research evidence**	**Additional considerations**
Moderate	High for MACE (pioglitazone and sulfonylureas); Moderate for all the other clinical outcomes	
**Values** Is there important uncertainty about or variability in how much people value the main outcomes?		
**Judgment**	**Research evidence**	**Additional considerations**
No important uncertainty or variability	No evidence of variability or uncertainty HbA1c, body weight, severe hypoglycemia, macrovascular complications and mortality are already considered among critical outcomes of the treatment of type 2 diabetes by scientific societies^4−6^	
**Balance of effects** Does the balance between desirable and undesirable effects favor the intervention or the comparison?		
**Judgment**	**Research evidence**	**Additional considerations**
Varies	The balance of effects favors metformin, GLP1 RA and SGLT2i over other classes of drugs, whereas it is unfavorable for sulfonylureas	
**Resources required** How large are the resource requirements (costs)?		
**Judgment**	**Research evidence**	**Additional considerations**
Varies	Low for metformin, pioglitazone, sulfonylureas, acarbose Moderate for other classes, higher for GLP1RA and insulin^18^	Some bioequivalent molecules could reduce direct costs for the most expensive approaches (i.e., insulin and GLP1RA)
**Certainty of evidence of required resources** What is the certainty of the evidence of resource requirements (costs)?		
**Judgment**	**Research evidence**	**Additional considerations**
High	Several good-quality studies explored this issue	
**Cost-effectiveness** Does the cost-effectiveness of the intervention favor the intervention or the comparison?		
**Judgment**	**Research evidence**	**Additional considerations**
Varies	The cost-effective evaluation depends on the drug used; comprehensive network meta-analysis exploring the economic implication of the different approaches are lacking, if we consider the large availability of options	
**Equity** What would be the impact on health equity?		
**Judgment**	**Research evidence**	**Additional considerations**
Probably no impact	Drugs recommended in the present guideline are already considered as first-and second-line treatment for patients without previous cardiovascular events in the principal guidelines^4−6, 52^	
**Acceptability** Is the intervention acceptable to key stakeholders?		
**Judgment**	**Research evidence**	**Additional considerations**
Probably yes	No specific evidence is available on this issue	
**Feasibility** Is the intervention feasible to implement?		
**Judgment**	**Research evidence**	**Additional considerations**
Probably yes	A large part of patients with type 2 diabetes in Italy is already treated with metformin, whereas GLP-1 RA and SGLT-2i are still relatively underutilized and sulfonylureas still prescribed, despite being less frequently than in the last years^19, 20^	

**5.3. Treatment with basal insulin**

Question: Should basal insulin analogues be preferred to NPH insulin in insulin-treated patients with type 2 diabetes?

**Table:** 

*Population*	People with type 2 diabetes
*Intervention*	Basal insulin analogues
*Comparison*	NPH insulin
*Outcome*	Hypoglycemia
*Setting*	Outpatient

**Relevant outcomes**.

**Table:** 

Outcome	Relevance (1–9)	Critical
Hypoglycemia	8	Yes
Quality of life	6	No
HbA1c	2	No
Body mass index	2	No
Ketosis	2	No

**RECOMMENDATION:**

**We recommend the use of basal insulin analogues, instead of NPH, for all patients with type 2 diabetes needing treatment with basal insulin.**

*Strength of the recommendation: strong. Quality of evidence: very low.*

**Justification.** A major body of evidence from randomized controlled trials supports the use of basal insulin analogues due to less risk of total and nocturnal hypoglycemia, with a trend toward reduction of severe hypoglycemia. Despite the treat-to-target design of the majority of RCT, a modest positive effect on HbA1c and FPG was observed (detemir e glargine U100). There are no available trials comparing newer basal insulin analogue formulations with NPH insulin. However, comparisons between glargine U100 and the newer formulations of insulin (degludec and glargine U300) show similar, and for same endpoints, more favorable effects for these latter two insulin formulations. Therefore, the recommendation to use basal insulin analogues, instead of NPH insulin, can be extended also to degludec and glargine U300.

The quality of available evidence is generally low, particularly due to the open-label design of the majority of the included trials and to the presence of heterogeneity.

Pharmacoeconomic studies showed that direct costs of drugs is generally increased with newer formulations despite the cost-effectiveness ratio generally suggest good value for money because of the implication in terms of both QALY and the effects on the risk of events, weight gain etc.; the availability of biosimilars contains the cost of out-of-patent insulin analogues.

***Subgroup considerations.*** No available evidence in patients aged over 75 years.

***Implementation.*** Long-acting analogues are already the standard of care. The prescription of NPH insulin should be strongly discouraged, with specific educational program for non-specialists, recommending its substitution with long-acting analogues.

***Assessment and monitoring***. The monitoring of adherence to guidelines on pharmacological treatment of type 2 diabetes can be implemented through the consultation of existing databases.

**Assessment**

**Table:** 

**Problem** Is the problem a priority?		
**Judgment**	**Research evidence**	**Additional considerations**
Yes	Hypoglycemia has a major impact on quality of life of insulin-treated patients^53−55^, and it represents a major obstacle for attaining desired glycemic goals Available data suggest that different long-acting insulin formulations are associated with different risk of hypoglycemia in type 2 diabetes^56−59^	
**Desirable Effects** How substantial are the desirable anticipated effects?		
**Judgment**	**Research evidence**	**Additional considerations**
Large	**Effects of basal insulin analogues vs NPH insulin** Total hypoglycemia: − 30% Nocturnal hypoglycemia: − 52% No significant effect on severe hypoglycemia: − 13%	No available comparisons with NPH insulin for newer basal insulin analogues (glargine U300, degludec) and aspart and lispro protamine
**Undesirable Effects** How substantial are the undesirable anticipated effects?		
**Judgment**	**Research evidence**	**Additional considerations**
Trivial	No relevant increase of any adverse event reported in clinical trials comparing basal insulin analogues with NPH insulin	
**Certainty of evidence** What is the overall certainty of the evidence of effects?		
**Judgment**	**Research evidence**	**Additional considerations**
Low	Low for all clinical outcomes	
**Values** Is there important uncertainty about or variability in how much people value the main outcomes?		
**Judgment**	**Research evidence**	**Additional considerations**
No important uncertainty or variability	No expected uncertainty or variability	
**Balance of effects** Does the balance between desirable and undesirable effects favor the intervention or the comparison?		
**Judgment**	**Research evidence**	**Additional considerations**
Favors the intervention	The balance of effects of using basal insulin analogues instead of NPH insulin is favorable for the reduction of total and nocturnal hypoglycemia	Despite treat-to-target design, modest, but significant, reduction of HbA1c and fasting plasma glucose (HbA1c: − 0.1% and FPG:-4 mg/dl), with no weight gain, was observed
**Resources required** How large are the resource requirements (costs)?		
**Judgment**	**Research evidence**	**Additional considerations**
Varies	Relevant direct costs^60^	The introduction of biosimilars reduced the average cost of out-of-patent long-acting insulin analogues
**Certainty of evidence of required resources** What is the certainty of the evidence of resource requirements (costs)?		
**Judgment**	**Research evidence**	**Additional considerations**
High	Several good-quality studies explored this issue	
**Cost-effectiveness** Does the cost-effectiveness of the intervention favor the intervention or the comparison?		
**Judgment**	**Research evidence**	**Additional considerations**
Probably favors the intervention	Pharmacoeconomic studies showed that direct costs of drugs is generally increased with newer formulations despite the cost-effectiveness ratio generally suggest good value for money because of the implication in terms of both QALY and the effects on the risk of events, weight gain etc.; the availability of biosimilars contains the cost of out-of-patent insulin analogues	The introduction of biosimilars reduced the average cost of out-of-patent long-acting insulin analogues, thus modifying the evaluation on cost-effectiveness ratio
**Equity** What would be the impact on health equity?		
**Judgment**	**Research evidence**	**Additional considerations**
Probably no impact	No impact expected (long-acting analogues are already the standard of care)^4, 20^	
**Acceptability** Is the intervention acceptable to key stakeholders?		
**Judgment**	**Research evidence**	**Additional considerations**
Probably yes	Long-acting analogues are already the standard of care in Italy^4, 20^	
**Feasibility** Is the intervention feasible to implement?		
**Judgment**	**Research evidence**	**Additional considerations**
Yes	Long-acting analogues are already the standard of care in Italy^4, 20^	

**5.4. Treatment with prandial insulin**

Question: Should prandial insulin analogues be preferred to human regular insulin in insulin-treated patients with type 2 diabetes?

**Table:** 

*Population*	People with type 2 diabetes
*Intervention*	Prandial insulin analogues
*Comparison*	Human regular insulin
*Outcome*	HbA1c, Hypoglycemia, Quality of Life, Patients’ preference
*Setting*	Outpatient

**Relevant outcomes**

**Table:** 

Outcome	Relevance (1–9)	Critical
Hypoglycemia	8	Yes
Quality of life	7	Yes
HbA1c	7	Yes
Patients’ preference	6	No
Body mass index	2	No
Ketosis	2	No

**RECOMMENDATION:**

**We suggest the use of prandial insulin analogues for patients with type 2 diabetes needing treatment with prandial insulin.**

*Strength of the recommendation: weak. Quality of evidence: very low.*

**Justification.** Low-quality evidence shows a better quality of life with analogues than with regular human insulin. Low quality of the studies included is mainly due to the open-label design, high heterogeneity and the relatively scarce number of patients enrolled.

The few pharmacoeconomic studies showed that rapid-acting insulin analogues in type 2 diabetes could be associated with a favorable balance of costs and effects due to the small effects on the hypoglycemic risk and the possible increase of quality of life.

***Subgroup considerations.*** None.

***Implementation.*** Short-acting analogues are already the standard of care^7,8^.

***Assessment and monitoring***. The monitoring of adherence to guidelines on pharmacological treatment of type 2 diabetes can be implemented through the consultation of existing databases^7,8^.

**Assessment**

**Table:** 

**Problem** Is the problem a priority?		
**Judgment**	**Research evidence**	**Additional considerations**
Yes	Hypoglycemia has a major impact on quality of life of insulin-treated patients^53−55^, and it represents a major obstacle for attaining desired glycemic goals In patients with type 1 diabetes, short-acting analogues provide a better control of postprandial glycemia associated with lower hypoglycemic risk in comparison with regular human insulin^61^. Some studies suggest that short-acting insulin analogues are associated with a lower hypoglycemic risk than human regular insulin and some metabolic advantages also in type 2 diabetes. However results are inconclusive and based on studies enrolling relatively few patients^62^	
**Desirable Effects** How substantial are the desirable anticipated effects?		
**Judgment**	**Research evidence**	**Additional considerations**
Small	**Effects of prandial insulin analogues vs human regular insulin** No significant effect on HbA1c and hypoglycemia Better quality of life scores for prandial analogues in one study^63^	
**Undesirable Effects** How substantial are the undesirable anticipated effects?		
**Judgment**	**Research evidence**	**Additional considerations**
Trivial	No relevant increase of any adverse event reported in clinical trials comparing prandial insulin analogues with human regular insulin	
**Certainty of evidence** What is the overall certainty of the evidence of effects?		
**Judgment**	**Research evidence**	**Additional considerations**
Very low	Very low for HbA1c; Low for all the other clinical outcomes	
**Values** Is there important uncertainty about or variability in how much people value the main outcomes?		
**Judgment**	**Research evidence**	**Additional considerations**
No important uncertainty or variability	No expected uncertainty or variability. HbA1c, hypoglycemia, and quality of life are already considered among critical outcomes of the treatment of type 2 diabetes by scientific societies^4−6^	
**Balance of effects** Does the balance between desirable and undesirable effects favor the intervention or the comparison?		
**Judgment**	**Research evidence**	**Additional considerations**
Probably favors the intervention	The balance of effects of using prandial insulin analogues instead of human regular insulin is favorable for the amelioration of quality of life, without any additional side effects	Short-acting analogues improve postprandial glucose control^62^
**Resources required** How large are the resource requirements (costs)?		
**Judgment**	**Research evidence**	**Additional considerations**
Varies	Relevant direct costs^60^	The introduction of biosimilars reduced the average cost of out-of-patent short-acting insulin analogues
**Certainty of evidence of required resources** What is the certainty of the evidence of resource requirements (costs)?		
**Judgment**	**Research evidence**	**Additional considerations**
Low	Few low-quality studies explored this issue	
**Cost-effectiveness** Does the cost-effectiveness of the intervention favor the intervention or the comparison?		
**Judgment**	**Research evidence**	**Additional considerations**
Probably favors the intervention	The few pharmacoeconomic studies showed that rapid-acting insulin analogues in type 2 diabetes could be associated with a favorable balance of costs and effects (small reduction of the hypoglycemic risk and amelioration of QoL)	The introduction of biosimilars reduced the average cost of out-of-patent long-acting insulin analogues, thus modifying the evaluation on cost-effectiveness ratio
**Equity** What would be the impact on health equity?		
**Judgment**	**Research evidence**	**Additional considerations**
Probably no impact	No impact expected (long-acting analogues are already the standard of care)^4, 20^.	
**Acceptability** Is the intervention acceptable to key stakeholders?		
**Judgment**	**Research evidence**	**Additional considerations**
Probably yes	Short-acting analogues are already the standard of care in Italy^4, 20^	
**Feasibility** Is the intervention feasible to implement?		
**Judgment**	**Research evidence**	**Additional considerations**
Yes	Short-acting analogues are already the standard of care in Italy^4, 20^	

**5.5. Treatment with continuous subcutaneous insulin infusion.**

Question: Should continuous subcutaneous insulin infusion be preferred in patients with type 2 diabetes not adequately controlled and treated with multiple daily injections?

**Table:** 

*Population*	People with type 2 diabetes
*Intervention*	Continuous subcutaneous insulin infusion
*Comparison*	Multiple daily injections
*Outcome*	HbA1c, Hypoglycemia, Quality of Life, Patients’ preference
*Setting*	Outpatient

**Relevant outcomes**

**Table:** 

Outcome	Relevance (1–9)	Critical
Hypoglycemia	8	Yes
Quality of life	8	Yes
HbA1c	8	Yes
Patients’ preference	6	No
Ketosis	4	No
Body mass index	2	No

**RECOMMENDATION:**

**The routine use of CSII in inadequately controlled patients with type 2 diabetes is not recommended.**

*Strength of the recommendation: weak. Quality of evidence: very low.*

**Justification.** There is no evidence of overall advantage of CSII over MDI, despite higher costs. The quality of available evidence is generally insufficient, particularly for “blinding procedures” due to the open-label design of the majority of the included trials.

No evidence available about pharmacoeconomic studies on CSII.

***Subgroup considerations.*** It is possible that CSII can have some clinical advantages in individual patients with type 2 diabetes on basal–bolus insulin requiring different supply of basal insulin during nocturnal time. CSII could provide advantages in those patients, but no specific subgroup analysis of patients with different profiles of fasting glucose has ever been performed in clinical trials.

***Implementation.*** None.

***Assessment and monitoring***. The monitoring of adherence to guidelines on pharmacological treatment of type 2 diabetes can be implemented through the consultation of existing databases.

**Assessment**

**Table:** 

**Problem** Is the problem a priority?		
**Judgment**	**Research evidence**	**Additional considerations**
Probably yes	Some studies suggest that continuous subcutaneous insulin infusion that have favorable effects in patients with type 1 diabetes^64, 65^, could have also some advantages in type 2 diabetes. However results are inconclusive and based on studies enrolling relatively few patients^56, 66, 67^	
**Desirable Effects** How substantial are the desirable anticipated effects?		
**Judgment**	**Research evidence**	**Additional considerations**
Trivial	**Effects of CSII versus MDI** ^64^ **:** No significant effect on HbA1c and hypoglycemia Inconclusive data on QoL. No available data on patients’ preference	CSII could have some advantages over MDI in specific subgroups of patients with type 2 diabetes (i.e., those with varying needs of basal insulin across the night), and some disadvantages in others (i.e., patients less accustomed to the use of complex technological devices)
**Undesirable Effects** How substantial are the undesirable anticipated effects?		
**Judgment**	**Research evidence**	**Additional considerations**
Trivial	No relevant increase of any adverse event reported in clinical trials comparing CSII with MDI	The complexity of infusion devices could theoretically increase the burden of therapy in some patients
**Certainty of evidence** What is the overall certainty of the evidence of effects?		
**Judgment**	**Research evidence**	**Additional considerations**
Very low	Very low for HbA1c and patients’ preference Low for severe hypoglycemia	
**Values** Is there important uncertainty about or variability in how much people value the main outcomes?		
**Judgment**	**Research evidence**	**Additional considerations**
No important uncertainty or variability	No expected uncertainty or variability. HbA1c, hypoglycemia, and quality of life are already considered among critical outcomes of the treatment of type 2 diabetes by scientific societies	
**Balance of effects** Does the balance between desirable and undesirable effects favor the intervention or the comparison?		
**Judgment**	**Research evidence**	**Additional considerations**
Does not favor either the intervention or the comparison	The balance of effects of using MDI instead of MDI is neutral	It is reasonable to believe that the use of CSII improves glycemic control in some patients (i.e., those with varying needs of basal insulin across the night), and it has a negative impact in others (i.e., patients less accustomed to the use of complex technological devices)
**Resources required** How large are the resource requirements (costs)?		
**Judgment**	**Research evidence**	**Additional considerations**
Large costs	Relevant direct costs	The introduction of newer products could reduce direct costs
**Certainty of evidence of required resources** What is the certainty of the evidence of resource requirements (costs)?		
**Judgment**	**Research evidence**	**Additional considerations**
No included studies	No evidence available on T2DM	
**Cost-effectiveness** Does the cost-effectiveness of the intervention favor the intervention or the comparison?		
**Judgment**	**Research evidence**	**Additional considerations**
Don't know	No evidence available on T2DM	
**Equity** What would be the impact on health equity?		
**Judgment**	**Research evidence**	**Additional considerations**
Probably reduced	The correct use of CSII requires a specific training and a careful follow-up, to be performed in specialist clinic with specific competence. This limits the accessibility of such treatment for many patients with type 2 diabetes	
**Acceptability** Is the intervention acceptable to key stakeholders?		
**Judgment**	**Research evidence**	**Additional considerations**
Don't know	No evidence available on T2DM	
**Feasibility** Is the intervention feasible to implement?		
**Judgment**	**Research evidence**	**Additional considerations**
Don't know	No evidence available on T2DM	

**6. Glucose monitoring.**

**6.1 Structured glucose monitoring**

Question: Should structured glucose monitoring be preferable in comparison with capillary glucose monitoring for diabetes control in patients with type 2 diabetes?

**Table:** 

*Population*	People with type 2 diabetes
*Intervention*	Structured glucose monitoring
*Comparison*	Capillary glucose monitoring
*Outcome*	HbA1c
*Setting*	Outpatient

**Relevant outcomes**

**Table:** 

Outcome	Relevance (1–9)	Critical
HbA1c	7	Yes
Hypoglycemia	6	No
Patients’ preference	4	No

**RECOMMENDATION:**

**We suggest to structure (with a pre-defined scheme of required tests) capillary blood glucose self-monitoring in the treatment of type 2 diabetes.**

*Strength of the recommendation: weak. Quality of evidence: very low.*

**Justification.** There are few low-quality trials, enrolling relatively few subjects, showing a small, but detectable, beneficial effects of structured glycemic monitoring on glycemic control. The quality of available evidence is low, and the limited sample size and some methodological issues in clinical trials downgrade the strength of the evidence. There is no expected difference in required resources.

***Subgroup considerations.*** There are few available data from randomized trials on the safety and efficacy of structured glucose in elderly patients. Patients with psychiatric disorders and cognitive impairment could benefit more from traditional educational prescription, often managed by caregivers.

***Implementation.*** The awareness of healthcare professionals of the benefits of structured glucose monitoring could be increased by specific educational programs. The inclusion of structured glucose monitoring among indicators of the quality of care for diabetes could be of help in increasing adherence to this recommendation.

***Assessment and monitoring***. The monitoring of this recommendation is problematic.

**Assessment**

**Table:** 

**Problem** Is the problem a priority?		
**Judgment**	**Research evidence**	**Additional considerations**
Yes	The use of capillary blood glucose self-monitoring is widespread among patients with type 2 diabetes. Determinations of blood glucose can be performed either randomly (based on patients' decision) or following a pre-defined (structured) scheme; some reports suggest that this latter modality may be preferable^68^	
**Desirable Effects** How substantial are the desirable anticipated effects?		
**Judgment**	**Research evidence**	**Additional considerations**
Small	**Effects of structured glucose monitoring** ^69^ **:** HbA1c: − 0.3%	
**Undesirable Effects** How substantial are the undesirable anticipated effects?		
**Judgment**	**Research evidence**	**Additional considerations**
Trivial	This issue was not explored	
**Certainty of evidence** What is the overall certainty of the evidence of effects?		
**Judgment**	**Research evidence**	**Additional considerations**
Very low	Very low for HbA1c	
**Values** Is there important uncertainty about or variability in how much people value the main outcomes?		
**Judgment**	**Research evidence**	**Additional considerations**
No important uncertainty or variability	No expected uncertainty or variability. HbA1c, hypoglycemia, and quality of life are already considered among critical outcomes of the treatment of type 2 diabetes by scientific societies^8−10^	
**Balance of effects** Does the balance between desirable and undesirable effects favor the intervention or the comparison?		
**Judgment**	**Research evidence**	**Additional considerations**
Probably favors the intervention	Small, but significant reduction of HbA1, with no adverse events	
**Resources required** How large are the resource requirements (costs)?		
**Judgment**	**Research evidence**	**Additional considerations**
Moderate savings	No additional direct costs. In some instances the intervention could determine a moderate savings	
**Certainty of evidence of required resources** What is the certainty of the evidence of resource requirements (costs)?		
**Judgment**	**Research evidence**	**Additional considerations**
Very low	There are few low-quality studies	
**Cost-effectiveness** Does the cost-effectiveness of the intervention favor the intervention or the comparison?		
**Judgment**	**Research evidence**	**Additional considerations**
Probably favors the intervention	The intervention could be cost-effective due to the reduction of HbA1c, with no additional required resources	
**Equity** What would be the impact on health equity?		
**Judgment**	**Research evidence**	**Additional considerations**
Probably no impact	No differences in costs and accessibility	
**Acceptability** Is the intervention acceptable to key stakeholders?		
**Judgment**	**Research evidence**	**Additional considerations**
Yes	No evidence available on T2DM	
**Feasibility** Is the intervention feasible to implement?		
**Judgment**	**Research evidence**	**Additional considerations**
Yes	Many patients in Italy are already on structured glucose monitoring^4, 20^	

**6.2 Subcutaneous continuous glucose monitoring**

Question: Should subcutaneous continuous glucose monitoring be preferable in comparison with capillary glucose monitoring for diabetes control in patients with type 2 diabetes treated with basal–bolus insulin schemes?

**Table:** 

*Population*	People with type 2 diabetes
*Intervention*	Subcutaneous continuous glucose monitoring
*Comparison*	Capillary glucose monitoring
*Outcome*	HbA1c; Hypoglycemia; Patients’ preference.
*Setting*	Outpatient

**Relevant outcomes**

**Table:** 

Outcome	Relevance (1–9)	Critical
HbA1c	8	Yes
Hypoglycemia	8	Yes
Patients’ preference	7	Yes

**RECOMMENDATION:**

**We do not suggest continuous glucose monitoring rather than self-monitoring blood glucose in patients with type 2 diabetes on basal–bolus insulin therapy.**

*Strength of the recommendation: weak. Quality of evidence: very low.*

**Justification.** Low-quality evidence suggests a small improvement of HbA1c associated with CGM; it is possible that CGM impairs quality of life in some patients. The use of CGM does not appear to be cost-effective.

***Subgroup considerations.*** No specific evidence is available for several subgroups that could have different results; in fact, younger age groups and subjects with higher HbA1c levels are more likely to benefit from the use of complex technology, whereas older patients could experience a more negative impact on quality of life.

***Implementation.*** None.

***Assessment and monitoring***. Adherence to this guideline can be assessed by estimating the proportion of patients at HbA1c target in existing databases^11,12^.

**Assessment**

**Table:** 

**Problem** Is the problem a priority?		
**Judgment**	**Research evidence**	**Additional considerations**
Probably yes	Several studies showed some beneficial effects of subcutaneous continuous glucose monitoring on health outcomes, including the reduction of HbA1c and the risk of hypoglycemia in type 1 diabetes^64^. Benefits observed in patients with type 1 cannot be automatically extended to those with type 2 diabetes, who differ for age, pathophysiology and comorbidities	
**Desirable Effects** How substantial are the desirable anticipated effects?		
**Judgment**	**Research evidence**	**Additional considerations**
Small	**Effects of structured glucose monitoring:** HbA1c: − 0.3% Hypoglycemia: no effect Patients’ preference: no available data Quality of life: either unchanged or reduced with CGM	
**Undesirable Effects** How substantial are the undesirable anticipated effects?		
**Judgment**	**Research evidence**	**Additional considerations**
Trivial	Patients’ self-reported quality of life is either unchanged or reduced with CGM, in comparison with SMBG	
**Certainty of evidence** What is the overall certainty of the evidence of effects?		
**Judgment**	**Research evidence**	**Additional considerations**
Very low	Very low for all critical outcomes	
**Values** Is there important uncertainty about or variability in how much people value the main outcomes?		
**Judgment**	**Research evidence**	**Additional considerations**
No important uncertainty or variability	No expected uncertainty or variability. HbA1c, hypoglycemia, and quality of life are already considered among critical outcomes of the treatment of type 2 diabetes by scientific societies^8−10^	
**Balance of effects** Does the balance between desirable and undesirable effects favor the intervention or the comparison?		
**Judgment**	**Research evidence**	**Additional considerations**
Probably favors the intervention	Small improvement of HbA1c in favor of CGM with no effect on the hypoglycemic risk. Possible deterioration of quality of life in some patients	The number and size of available trials are not sufficient for reliable subgroup analyses. It is possible that benefits are greater, and detrimental effects smaller, in specific subgroups of patients
**Resources required** How large are the resource requirements (costs)?		
**Judgment**	**Research evidence**	**Additional considerations**
Trivial	No relevant additional direct costs. Some studies show high direct costs with relevant heterogeneity depending from the setting studied	
**Certainty of evidence of required resources** What is the certainty of the evidence of resource requirements (costs)?		
**Judgment**	**Research evidence**	**Additional considerations**
Moderate	There are some good-quality studies on this issue	
**Cost-effectiveness** Does the cost-effectiveness of the intervention favor the intervention or the comparison?		
**Judgment**	**Research evidence**	**Additional considerations**
Probably favors the intervention	The intervention could be cost-effective due to the reduction of HbA1c, with no additional required resources	Some patient’s characteristics or the glucose control could modify the judgment on cost-effectiveness
**Equity** What would be the impact on health equity?		
**Judgment**	**Research evidence**	**Additional considerations**
Probably reduced	No specific evidence on this issue	Elderly subjects have greater difficulties in acquiring technological skills^21^
**Acceptability** Is the intervention acceptable to key stakeholders?		
**Judgment**	**Research evidence**	**Additional considerations**
Probably yes	No specific evidence available on this issue	It is possible that some subgroups of patients (e.g., those with advanced age) may find the use of this technology more intrusive
**Feasibility** Is the intervention feasible to implement?		
**Judgment**	**Research evidence**	**Additional considerations**
Probably yes	No specific evidence available	The instruction of a large number of patients to the use of this technology could represent a relevant burden for specialist diabetes care units

**Fig. 1:**
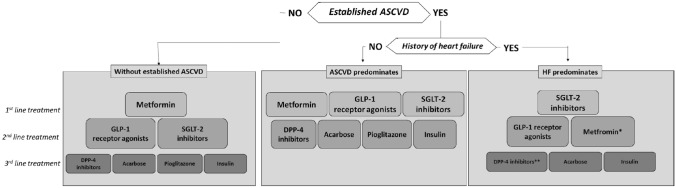
Therapeutic algorithm for the pharmacological treatment of type 2 diabetes
